# Establishment and application of a multiplex qPCR panel for multi-host surveillance of tick-Borne pathogens

**DOI:** 10.1080/22221751.2026.2731498

**Published:** 2026-09-12

**Authors:** Jing Li, Zhen Wang, Jing Li, Xinting Meng, Jiaxun Zhang, Wenjin Mao, Yan Hai, Ying Zhang, Xiangxiang Sun, Jin Che, Xiaofang Ma, Biao Duan, Aiping Qin, Jie Liu

**Affiliations:** a School of Public Health, Qingdao University, Shandong, People’s Republic of China; b Department of Laboratory Medicine, The Affiliated Hospital of Qingdao University, Qingdao, People’s Republic of China; c Department of Biosafety, General Center for Disease Control and Prevention of Inner Mongolia Autonomous Region, Hohhot City, People’s Republic of China; d Department of Vector-Borne Disease, Center for Disease Control and Prevention of Xilingol League, Xilinhaote, People’s Republic of China; e Key Laboratory of Animal Tuberculosis, Ministry of Agriculture and Rural Affairs (Reference Laboratory for Animal Tuberculosis), China Animal Health and Epidemiology Center, Qingdao, People’s Republic of China; f State Key Laboratory for Animal Disease Control and Prevention, Key Laboratory of Veterinary Parasitology of Gansu Province, Chinese Academy of Agricultural Sciences, Lanzhou Veterinary Research Institute, Gansu, People’s Republic of China; g Institute of Disinfection and Vector Control, Qingdao Municipal Center for Disease Control and Prevention, Qingdao, People’s Republic of China; h Plague Prevention and Control Division, Yunnan Institute of Endemic Diseases Control and Prevention, Dali, People’s Republic of China; i Department of Respiratory Disease, National Institute for Communicable Disease Control and Prevention, Chinese Center for Disease Control and Prevention, Beijing, People’s Republic of China

**Keywords:** Multiplex qPCR, tick-borne pathogen, epidemiological surveillance, acute febrile illness, molecular diagnosis

## Abstract

Tick-borne diseases pose significant public health threats. Non-specific symptoms and co-infections present diagnostic challenges for conventional methods. To address this, we developed the tick-borne pathogen (TBP) Panel, utilizing eight separate multiplex qPCR assays to simultaneously detect 25 TBPs (7 bacteria, 14 viruses, 4 parasites). Optimal Quantification Cycle (Cq) cut-offs were established using nested/semi-nested PCR amplicon sequencing, and digital PCR as reference standards. The Panel was then deployed for screening 2,333 ticks, 59 rodents, and 553 febrile patients. The Panel demonstrated robust analytical performance, achieving limits of detection (LOD_99%_) being 3.5–131 copies/reaction of plasmid, 10^2^–10^3^ copies/mL of blood and 10^3^–10^4^ copies/tick pool. This study established a tiered determination framework (Cq ≤ 36.6 as positive; Cq 36.6–39.6 requiring dual-replicate confirmation), yielding an overall AUC of 0.979, with 96.5% sensitivity and 96.5% specificity. Screening revealed a diverse pathogen landscape, identifying a total of 14 pathogens in 16.9% of the ticks (395/2,333), revealing a diverse geographical distribution. Five pathogens were identified in 7.9% of AFI patients (25/317), including Severe Fever with Thrombocytopenia Syndrome Virus (SFTSV) and *Ehrlichia* spp., which were found circulating in local ticks. In the FUO cohort, a single case of *Rickettsia* spp. was identified. Rodent surveillance identified four pathogens, predominantly *Bartonella* spp. (67.8%). In conclusion , the rigorously validated TBP Panel enables rapid etiological identification of undifferentiated febrile illnesses, serving as a high-throughput tool that bridges large-scale surveillance with clinical diagnostics to close critical gaps in tick-borne disease management.

## Introduction

Ticks are globally recognized as the second most important vector after mosquitoes, transmitting a diverse range of pathogens through their blood-feeding activities, including bacteria, viruses, and protozoa [1, 2]. Tick-borne diseases (TBDs) pose a substantial public health threat, exemplified by the estimated 476,000 Lyme disease cases diagnosed and treated annually in the US alone [3]. In nature, wild rodents act as primary reservoir hosts, sustaining the circulation of numerous tick-borne pathogens (TBPs) by providing blood meals for immature ticks [4, 5]. Furthermore, ecological shifts and human encroachment into natural habitats have increased human contact with vectors, thereby elevating the risk of infection with multiple TBPs [6]. Consequently, mitigating TBDs urgently requires broad-spectrum screening technologies coupled with comprehensive epidemiological surveillance.

However, diagnosing and monitoring TBPs remains a formidable challenge. Clinically, non-specific febrile symptoms, coupled with low bloodstream pathogen loads and high pathogen diversity, frequently drive misdiagnosis [2, 7, 8]. While conventional methods such as culture and serology remain foundational for definitive diagnosis, their clinical utility is hindered by prolonged turnaround times and low yield for early stage detection [9–11]. Conversely, standard molecular techniques, in particular well-established PCR-based methods, offer high sensitivity and specificity for precise TBP identification [12, 13]. However, relying on single-target assays to sequentially screen numerous pathogens during syndromic clinical testing or large-scale ecological surveillance is labor-intensive, highly inefficient, and rapidly depletes precious samples.

While metagenomic next-generation sequencing (mNGS) excels at unbiased pathogen discovery, its clinical application is hindered by high costs, prolonged turnaround times, and intensive bioinformatics demands [14, 15]. These operational barriers are compounded by technical vulnerabilities like contamination and limited sequencing depth, which frequently cause low-load bloodstream infections to escape detection [16, 17]. Extending this technology to vector surveillance introduces another critical blind spot: the need for separate DNA and RNA library preparations severely risks omitting co-infecting pathogens [18, 19]. Consequently, targeted, highly sensitive, and multiplexed methods like qPCR remain essential for both accurate clinical diagnosis and ecoepidemiological surveillance.

To address these challenges, based on national pathogenic microorganism catalogues and epidemiological evidence, we systematically integrated a spectrum of highly prevalent or emerging zoonotic pathogens that are posing public health threats in China [2, 20]. Building upon this, we ultimately developed and validated a high-throughput multiplex TaqMan qPCR panel capable of detecting 25 key TBPs. Using this method, we profiled pathogen distribution across febrile patients, ticks, and rodents. Ultimately, this dual-purpose platform integrates clinical diagnostics with ecological surveillance, providing a practical tool for the clinical management and regional control of TBDs.

## Materials and methods

### Samples collection

Between July 2023 and August 2025, 2,333 ticks were collected across 18 Chinese provinces via vegetation flagging and removal from domestic animals. Ticks were morphologically identified and aggregated into 225 pools by species, site, and life stage (1–10 engorged, 10–20 adults/nymphs, or 50–80 larvae; Table S1). Species-level identification of each pool primarily relied on the amplification of cytochrome c oxidase subunit I (COI) gene, with 16S rRNA serving as a complementary marker when COI amplification failed or lacked resolution (e.g. for *Hyalomma* spp.) as previously described (Table S2) [21, 22].

Whole blood samples (n = 553) were obtained from two distinct cohorts: a prospective acute febrile illness (AFI) cohort (n = 317); Laoshan Affiliated Hospital of Qingdao University, August 2023–July 2024; and a retrospective fever of unknown origin (FUO) surveillance cohort (n = 236; Inner Mongolia Center for Disease Control and Prevention, March and December 2021). Detailed inclusion/exclusion criteria are provided in Table S3.

In September 2022, a convenient set of 59 rodents were collected in Jinghong city, Yunnan Province, and morphologically identified. Liver tissues were subsequently harvested.

All samples were transported on cold chain and stored at −80 °C until testing.

### Total nucleic acid extraction

Ticks were homogenized in liquid nitrogen, while rodent livers underwent mechanical disruption (OMNI Bead Ruptor 24 Elite; 6 m/s, 4× 20s), 56 °C incubation (10 min), and centrifugation. All samples were spiked with MS2 bacteriophage (1 ×   10^6^ copies/Sample) to monitor extraction/amplification efficiency, alongside batch-specific negative controls.

Nucleic acids were extracted using two distinct kits from Bioer Technology (Hangzhou, China): automated magnetic bead extraction (MAGABio Virus DNA/RNA Maxi Kit II) for 2 mL AFI whole blood, and manual column-based extraction (Biospin Virus DNA/RNA Extraction Kit) for 200 μL aliquots of FUO blood, tick pools, and rodent livers.

### TBP panel design

A comprehensive set of 25 priority tick-borne pathogens (7 bacteria, 14 viruses, 4 parasites), detailed in Table 1, was selected based on their prevalence or emergence in China [2, 23]. These 25 pathogens, plus MS2 external control, were formulated into eight multiplex reactions (Table S4). Primers and probes were adapted from published literatures whenever feasible. De novo designs using Primer3 underwent in silico validation to ensure specificity, including the cross-reactivity between targets in the same reaction. This involved screening for secondary structures (e.g. dimers, hairpins) with Multiple Primer Analyzer [24] and OligoAnalyzer™ Tool (Integrated DNA Technologies, Coralville, IA, USA), followed by a BLASTn search against the NCBI database. Additionally, the oligo length and GC contents were adjusted for a tight Tm distribution for all the primers and probes, respectively, aiming to apply a universal annealing temperature. All oligonucleotides were synthesized by Sangon Biotech (Wuhan, China).

**Table 1.** Analytical performance of TBP Panel for simultaneous detection of tick-borne pathogens.

Assay	Pathogen	Limit of detection 99% (99% CI)		Spec, %	Sens, %
		Blood (Copies/ mL)	Ticks (Copies / pool)	(TN / FP)	(TP / FN)
1	BJNV	1097.8	23607.2	100	100
		(866.9–1827.2)	(18188.2–37005.4)	(24 / 0)	(6/0)
	JMTV	1049.1	25061.3	100	NA
		(847.2–1641.9)	(19308.4–39284.2)	(30/0)	(0/0)
	XCV	798.9	25399.7	96.8	NA
		(674.4–1316.1)	(19699.7–39237.4)	(30/1)	(0/0)
2	*Bartonella* spp.	1025.5	28718.1	93.3	66.7
		(849.4–1639.3)	(21668.1–48062.2)	(28/2)	(2/1)
	*Borrelia* spp.	1088.9	31189.2	89.5	100
		(881.2–1717.3)	(23341.9–53189.2)	(17/2)	(13/0)
	TBEV	1241.7	32388.2	100	NA
		(959.3–2158.5)	(26149.1–50707.2)	(30/0)	(0/0)
	YEZV	1156.4	35088.3	100	NA
		(919.7–1902.8)	(27035.3–55931.2)	(30/0)	(0/0)
3	ALSV	1108.9	21223.5	100	100
		(876.2–1775.8)	(15174.0–40733.7)	(29/0)	(1/0)
	*F. tularensis*	944.72	9094.9	100	NA
		(589.7–3733.1)	(6352.4–21110.8)	(30/0)	(0/0)
	SFTSV	1031.5	18468.7	90	100
		(832.9–1614.2)	(13232.6–35465.7)	(27/3)	(3/0)
4	*A. phagocytophilum*	728.6	18090.3	100	100
		(599.3–1172.1)	(13127.7–33447.4)	(27/0)	(3/0)
	CCHFV	910.4	17099.0	100	NA
		(739.0–1407.2)	(12408.4–31614.7)	(30/0)	(0/0)
	*Rickettsia* spp.	820.9	8888.4	83.3	83.3
		(666.4–1286.2)	(6328.4–19185.3)	(15/4)	(15/2)
5	*C. burnetii*	1093.0	17233.3	100	100
		(869.3–1798.5)	(12369.2–32950.8)	(15/0)	(15/0)
	*Ehrlichia* spp.	1066.8	18562.2	84.2	100
		(823.9–1814.9)	(12518.7–44935.2)	(17/3)	(14/0)
	*T*. *gondii*	1083.8	11279.69	100	NA
		(839.4–1838.0)	(7667.3–27935.0)	(30/0)	(0/0)
6	SGLV	1233.7	23026.0	93.8	NA
		(962.3–2187.8)	(17558.0–36901.3)	(30/2)	(0/0)
	*Hepatozoon* spp. / *Theileria* spp.	1286.2	22943.0	93.8	100
		(971.6–2379.9)	(16023.8–47462.9)	(15/1)	(15/0)
7	NSDV	1207.7	34143.5	88.9	100
		(938.7–2081.3)	(24803.2–61810.7)	(24/3)	(6/0)
	TAMV	1044.2	22129.6	100	NA
		(855.8–1640.6)	(17085.4–34774.4)	(30/0)	(0/0)
	WELV	967.1	29044.3	96.8	NA
		(793.9–1535.4)	(23543.2–42027.8)	(30/1)	(0/0)
8	*Babesia* spp.^†^	767.9	9435.9	100	100
		(620.2–1293.2)	(6718.2–20367.4)	(14/0)	(16/0)
	*Babesia duncani*^†^	863.6	8949.5	100	NA
		(683.7–1379.4)	(7151.2–13795.0)	(30/0)	(0/0)
	*Babesia microti*^†^	2909.7	19502.4	100	100
		(2113.9–5618.3)	(14857.3–31926.5)	(28/0)	(2/0)
	NoMV	1266.1	9416.8	100	80
		(991.6–2157.4)	(7169.0–16615.9)	(26/0)	(4/1)
	TcTV-1	1049.1	9415.5	100	NA
		(847.2–1641.8)	(6794.6–19356.9)	(30/0)	(0/0)

**Abbreviations**: TN: True Negative; FP: False Positive; TP: True Positive; FN: False Negative; NA: Not Applicable. Sensitivity could not be calculated because no true-positive clinical or vector samples were available in the validation panels. For these pathogens, analytical sensitivity was estimated solely based on synthetic plasmid standards.

**Note**: ^†^The genus-level *Babesia* spp. assay does not cross-detect *B. duncani* and *B. microti* as they represent three distinct groups within the genus. Although *Babesia* spp. and *B. duncani* share the FAM channel in the final panel, distinct fluorophore-labeled probes were temporarily used during validation to prevent signal overlap, enabling independent evaluation of sensitivity and specificity while ruling out cross-detection.

### Optimization of reaction conditions

qPCR conditions were established through a sequential optimization strategy. Singleplex primer and probe concentrations were initially evaluated using gradients of 400–1000 nM (100 nM increments) and 150–350 nM (50 nM increments), respectively. These assays were then multiplexed, and a uniform annealing temperature was selected from a thermal gradient of 58, 60, and 62 °C.

### qPCR reaction

Each 10 μL qPCR reaction containing 2 μL of plasmid template and 2 μL of HiScript III U + One Step qRT-PCR Probe Master Mix (Vazyme, Nanjing, China), was run on an Applied Biosystems 7500 instrument (Thermo Fisher Scientific, Waltham, MA, USA). The cycling conditions consisted of reverse transcription (RT) at 55 °C for 15 min and denaturation at 95 °C for 30 s, followed by 45 cycles of 95 °C for 10 s and 60 °C for 60 s. To maintain consistent reaction conditions, the same One Step RT-qPCR protocol was used when plasmid constructs were amplified.

### Analytical performance

A synthetic plasmid encompassing all 25 targets [25] served as the pooled positive control (Sangon Biotech, China). Inclusivity was verified using 97 strains representing 25 intended target pathogens (Table S5). Analytical specificity (exclusivity) was confirmed against 94 strains representing 47 non-target pathogens (Table S6). Pearson coefficient (*R*^2^), and the amplification efficiency (E) were determined using 10-fold serial dilutions (1 × 10^1^–1 × 10^5^ copies/reaction).

The 99% limit of detection (LOD_99%_) was established using negative tick homogenates and human EDTA whole blood spiked with plasmid constructs prior to nucleic acid extraction. Six 10-fold serial dilutions, with concentrations ranging from 1 × 10^1^ to 1 × 10^6^ copies/sample, were extracted and tested in eight replicates. The LOD_99%_ and associated confidence intervals were calculated via probit regression, defining Cq ≥ 40 as negative.

Assay precision (coefficient of variation, CV) was evaluated at two concentrations (1 × 10^5^ and 1 × 10^6^ copies/reaction). Intra- and inter-assay CVs were calculated from 20 replicates within a single run and across 20 independent runs, respectively. All procedures strictly adhered to MIQE guidelines (Table S7) [26].

### Confirmatory nested PCR and dPCR

To validate the TBP Panel, results were compared against nested/semi-nested PCR (nPCR/snPCR) coupled with amplicon sequencing. Specifically, for nPCR/snPCR sample selection, 30 tick pools were randomly selected for each pathogen target, yielding a total of 720 independent evaluations. First- and second-round PCR were performed in 25 µL reaction volumes (each incorporating 2 µL of template) using the AccurSTART OneStep RT-PCR Kit or 2× Taq Plus Master Mix (Vazyme, China). Target-specific thermal cycling conditions are detailed in Table S8.

For qPCR-positive but sequencing-negative samples, the QIAcuity digital PCR (dPCR) system (QIAGEN) served as the adjudicating reference. The eight multiplex dPCR assays were assembled in 8.5 K 24-well nanoplates with a 12 µL total volume per reaction, comprising: 5.88 µL of template, 3 µL of 4× OneStep Advanced Probe Master Mix, 0.12 µL of 100× RT Mix, 1.5 µL of Enhancer GC, 0.5 µL of primer/probe mixture (using the optimized qPCR concentrations detailed in Table S4), and 1 µL of 0.1× *XbaI* restriction enzyme to optimize template partitioning. nPCR/snPCR and dPCR thermal cycling was performed strictly according to the manufacturer’s instructions.

Absolute quantification was calculated via the QIAcuity Suite using Poisson statistics. Positivity required clear partition segregation, ≥ 3 positive partitions, and a concentration exceeding the limit of blank (LOB; established from 20 no-template controls).

### Application of the TBP panel

Reactions utilized 2 µL of template for tick pool/rodent samples and 5 µL for human samples. Human screening employed a 5-in-1 pooling strategy; presumptively positive pools (Cq ≤ 40) were de-pooled for individual testing. Final positivity was confirmed via individual singleplex qPCR (Cq ≤ 40) or nPCR/snPCR coupled with Sanger sequencing.

### Data analysis

Statistical analyses were performed using R software (version 4.6.0), unless otherwise specified. Categorical variables (e.g. gender and symptoms) were summarized as counts and percentages (n, %), while continuous variables (e.g. age) were presented as medians with interquartile ranges (IQR). The non-parametric Mann–Whitney U test was employed to compare Cq value distributions between dPCR-confirmed positive and negative groups using the base stats package.

The diagnostic performance of the Panel was evaluated against amplicon sequencing, with dPCR selectively verifying positive samples. Outcomes were classified based on these reference methods (True Positive: Panel+/ sequencing +  or dPCR+; False Positive: Panel+/ sequencing- or dPCR-; False Negative: Panel-/sequencing+; True Negative: Panel-/sequencing-). ROC curve analysis was conducted using SPLS software (version V2.0; https://www.spls.cn) to determine the area under the curve (AUC) and its 95% confidence interval (CI) with DeLong’s test, and the optimal Cq cutoff with Youden’s index. Sensitivity, specificity, and likelihood ratios (LR + and LR-) were calculated to assess clinical utility. The 95% CIs for these proportions were calculated using the standard Wald method when normal approximation criteria were met (the denominator n ≥   30, and expected frequencies np ≥   5 and n(1-p) ≥   5). Otherwise, the Wilson score interval method was applied to ensure robust estimation. Probit regression was applied to determine the 99% limit of detection (LOD_99_%). Individual tick prevalence was calculated using maximum likelihood estimation (MLE) via the PooledInfRate software [27]. The graphic abstract was created with BioGDP.com [28].

## Results

### Development and optimization of the multiplex qPCR panel

Published or newly designed assays targeting genome sequences that were previously identified as unique for interrogated pathogens were formulated into a multiplex assay. The qPCR conditions were optimized and the finalized primer and probe concentrations are detailed in Table S4. A universal, standardized annealing temperature of 60°C was adopted for all reactions. Notably, the Cq differences between the multiplex and singleplex formats for all targets were within one Cq unit (ΔCq ≤   1, Table S9), indicating no significant target interference that compromises amplification efficacy.

### Analytical performance

The Panel demonstrated excellent linearity over a dynamic range of 1 ×   10^1^–1 ×   10^5^ copies/reaction. For all targets, the linear regression yielded *R*^2^ greater than 0.99. The calculated amplification efficiencies ranged between 86.29% and 106.35%, as detailed in Table S10. The test results confirmed 100% inclusivity across all 25 interrogated pathogens (Table S5), as well as 100% analytical specificity with no cross-reactivity among the 47 non-target pathogens (Table S6).

The LOD_99%_ for the Panel was determined in synthetic plasmid and spiked matrices. The Panel demonstrated consistent analytical sensitivity for targets, with LOD_99%_ values ranging from 3.5 to 1.3× 10^2^ copies/reaction for plasmid standard (Table S11), 7.3 × 10^2^–2.9 × 10^3^ copies/mL of whole blood, and 8.9 × 10^3^–3.5 × 10^4^ copies/pool in the tick matrix (Table 1).

The Panel demonstrated excellent precision and reliability. The intra-assay CVs, namely repeatability, were between 0.29% and 1.52% for the two concentrations tested. The inter-assay CVs, namely reproducibility, ranged from 0.72% to 1.71%. As detailed in Table S10, all CVs were well below 2.0%.

### Diagnostic performance of the TBP panel

**Figure 1.**
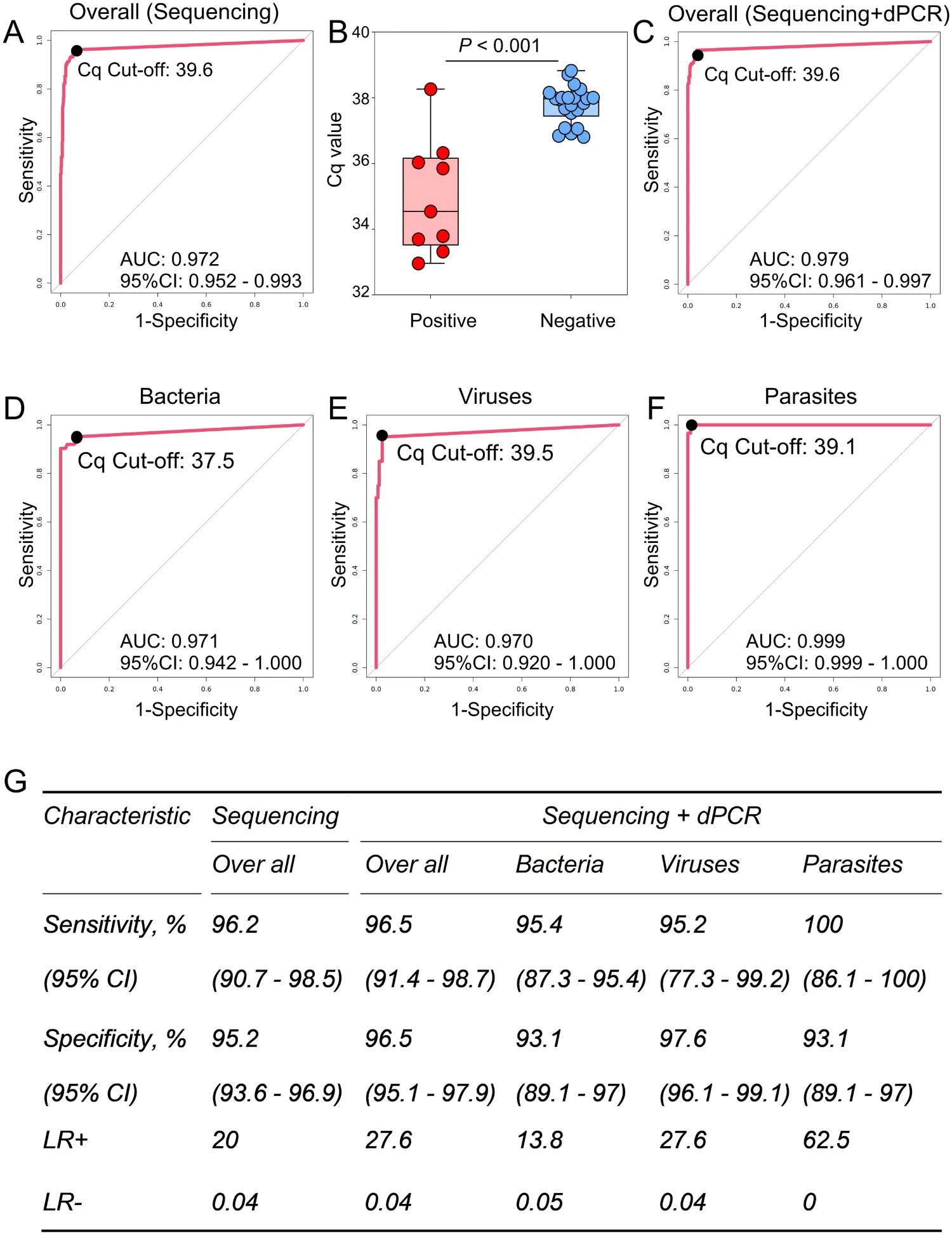
Diagnostic performance and analytical validation of the TBP Panel. (A) Overall receiver operating characteristic (ROC) curve using sequencing as the reference standard. (B) Distribution of Cq values comparing dPCR-positive and dPCR-negative samples. (C) Overall ROC curve utilizing a combined reference standard of sequencing and dPCR. (D-F) Subgroup ROC curves stratified by pathogen type, illustrating performance for bacteria (D), viruses (E), and parasites (F). (G) Summary table of pooled diagnostic performance metrics. Note: *PPA, Positive Percent Agreement; NPA, Negative Percent Agreement; LR+, Positive Likelihood Ratio; LR-, Negative Likelihood Ratio.

Diagnostic performance was evaluated against nPCR/snPCR sequencing through 720 independent evaluations (30 per pathogen from 225 tick pools; Table 1). Based on ROC curve analysis, an optimal Cq cut-off of 39.6 (AUC: 0.972) (Figure 1(A)). However, discordant results occurred in 4.9% (35/720) of evaluations. While concordance was high for pathogens like *Babesia* spp. and BJNV, discordance predominantly involved *Rickettsia* spp. and *Ehrlichia* spp. (Table 1 and S12). Specifically, 31 qPCR-positive but sequencing-negative samples exhibited late-cycle amplification with a median Cq of 37.7 (IQR: 36.6–38).

As shown in Figure 1(B) and Table S12, dPCR confirmed 29.0% (9/31) of late-cycle specimens as true positives. Cq values for this dPCR-positive group were significantly lower than the negative group (median 34.5 [IQR: 33.7–36] vs. 38.0 [IQR: 37.6–38.0]; *P* <   0.001). Notably, 100% PPA was observed for all samples with a Cq ≤   36.6. Using this calibrated standard alongside the Cq 39.6 cut-off refined overall sensitivity and specificity to 96.2% and 96.5%, respectively (Figures 1(C) and 1(G)). Furthermore, stratified ROC analyses across bacteria, viruses, and parasites yielded excellent AUCs (0.97–0.999; Figures 1(D)–1(F)), with the parasite group exhibiting the highest diagnostic performance (LR + 62.5, LR- 0; Figure 1(G)).

### Pathogen detection in ticks

We deployed the Panel to screen 2,333 ticks across five classic regions defined by integrated climatic, ecological, and zoogeographical characteristics (23), including Northeastern, Northern, Central, and Southern China, as well as the Inner Mongolia-Xinjiang region. Notably, eastern Inner Mongolia falls within the Northeastern region, whereas its central and western parts belong to the Inner Mongolia-Xinjiang region.

**Figure 2.**
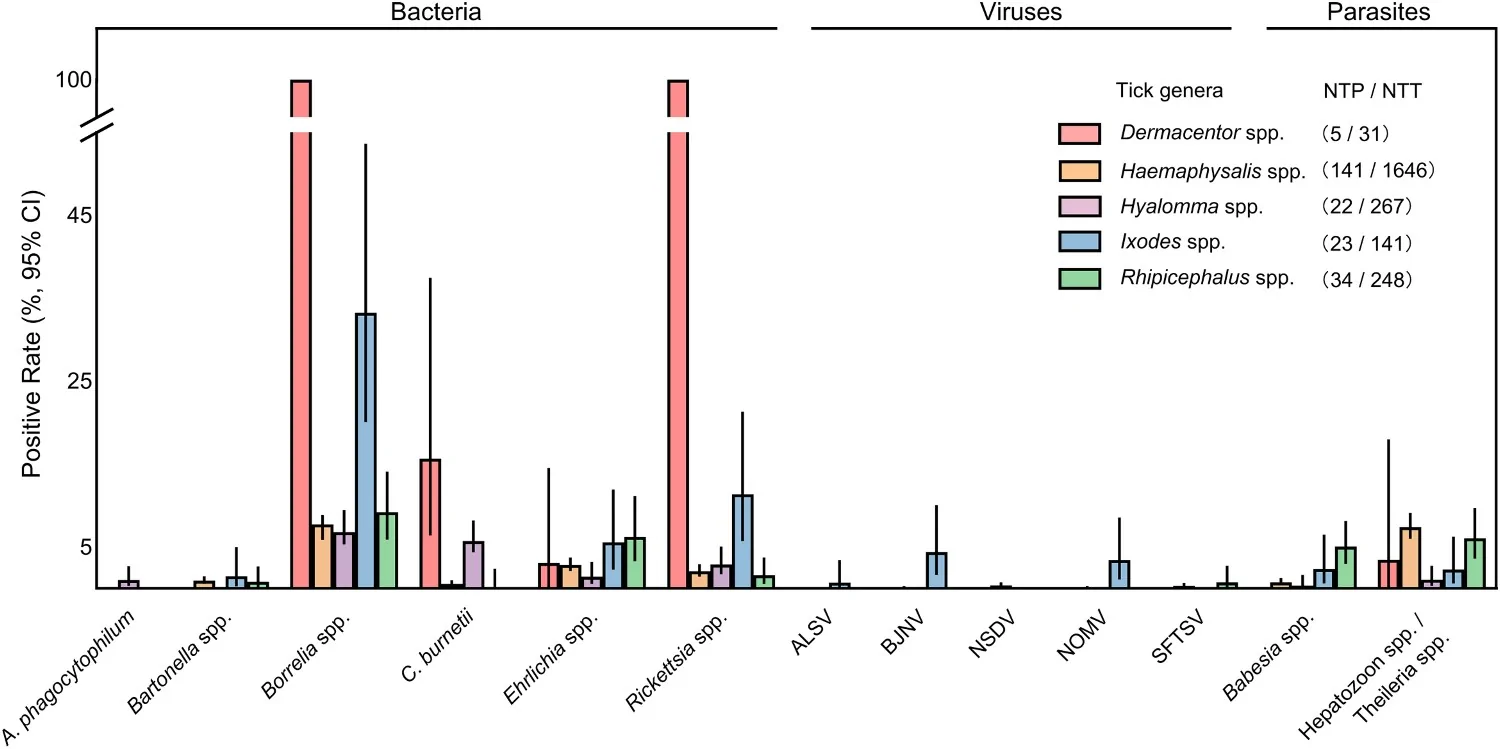
Pathogen prevalence across five tick genera collected from five zoogeographical regions in China. The chart displays the prevalence of 14 pathogens detected by the TBP Panel. Ticks were pooled by species. The x-axis represents specific pathogens, with colored bars distinguishing the five tick genera. Prevalence (y-axis) was calculated via maximum likelihood estimation (MLE) using PooledInfRate, with error bars indicating 95% CI. The number of tested ticks (NTT) and the number of tick pools (NTP) per genus are annotated.

Ticks spanned five genera, predominantly *Haemaphysalis* spp. (n = 1,646) and *Hyalomma* spp. (n = 267) (Figure 2, Table S1). MS2 internal controls achieved a 100% validity rate across all tick pools (mean Cq: 29.4 ± 1.0); mild Cq delays (3–5 cycles) in 11 engorged tick pools (4.9%) remained within the valid range (Cq < 35), and stable amplification was fully restored upon 1:5 dilution. Overall, 14 pathogens were detected, yielding an 18.5% infection rate. Positivity varied by genus, ranging from 7.4% in *Hyalomma* spp. to 37.5% in *Ixodes* spp. Notably, all *Dermacentor* pools (n = 5) tested positive for both *Borrelia* and *Rickettsia*, though this 100% rate requires cautious interpretation due to the limited sample size (Figure 2, Table S1).

Geographically, pathogen distribution exhibited distinct vector-region specificities (Table S1). Bacteria predominated, with *Borrelia* (17.2%) and *Ehrlichia* (19.3%) peaking in Southern and Central China, respectively. The Inner Mongolia-Xinjiang region featured notable detection of multiple bacteria, including *Borrelia*, *C. burnetii,* and *Rickettsia*. Viruses remained undetected within the Southern and Inner Mongolia-Xinjiang. However, ALSV (0.9%), BJNV (8.4%), and NOMV (6.6%) were exclusively found in Northeastern *Ixodes*. In contrast, SFTSV (0.3%) and NSDV (0.4%) were detected in Northern *Haemaphysalis*, while SFTSV (1.5%) was also detected in Central *Rhipicephalus*. Among ubiquitous protozoa, the *Hepatozoon*/*Theileria* complex predominated in Northern China’s *Haemaphysalis* (7.4%) and *Rhipicephalus* (6.1%), while *Babesia* peaked in *Rhipicephalus* (5.1%).

### Pathogen detection in febrile patients

**Figure 3.**
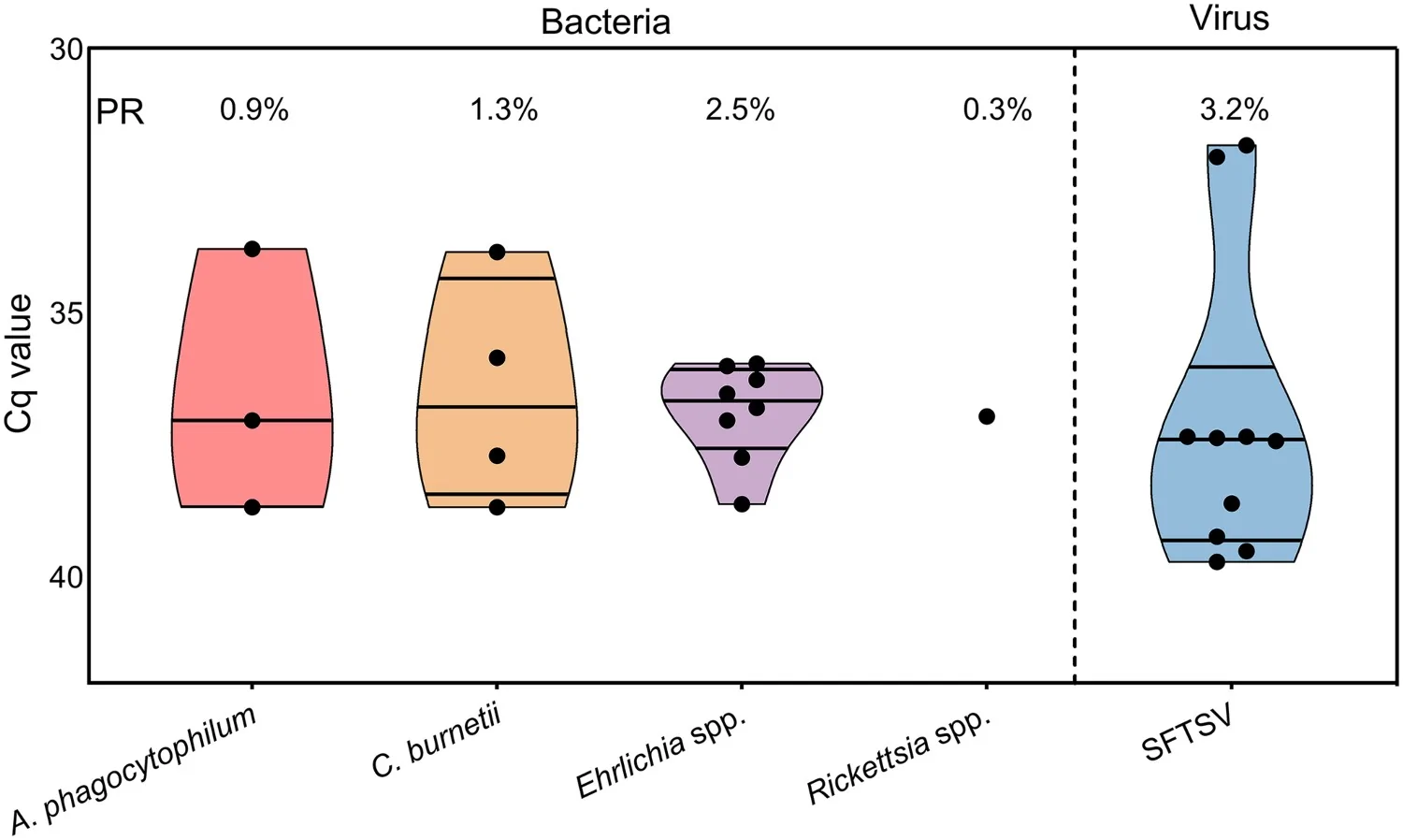
Distribution of Cq values for tick-borne pathogens detected in acute febrile illness patients from Qingdao. The positive rate (PR, %) is annotated above each respective plot.

Demographic and clinical profiles for the 317 Qingdao AFI and 236 Inner Mongolia FUO patients are detailed in Table S3. The Qingdao cohort primarily presented with undifferentiated fever and mild respiratory symptoms (35.3%). All AFI blood samples achieved a 100% validity rate for extraction and MS2 internal controls (mean Cq: 27.4 ± 1.4). Two samples (0.6%) with initial extraction failures were successfully re-extracted. The Panel identified pathogens in 25 AFI patients (7.9%; predominantly male [60.0%], median age 39), who typically exhibited fatigue/malaise or cough (both 30.8%). Primary agents were SFTSV (3.2%, 10/317) and *Ehrlichia* (2.5%, 8/317) (Figure 3), including one *A. phagocytophilum*/*Ehrlichia* co-infection. Notably, three low-titer samples (Cq ≥ 40) detected by the multiplex panel tested negative via corresponding singleplex assays.

In contrast, the Inner Mongolia FUO cohort comprised mainly farmers and herdsmen (69.5%) with frequent animal contact (75.4%), overwhelmingly presenting with myalgia or arthralgia (95.3%). Extraction and internal controls demonstrated a 100% validity rate across this cohort, with a mean Cq of 29.2 ± 1.5. However, identifying only a single *Rickettsia* spp. infection (Cq 33.3) in an elderly male farmer from Ulanqab, Inner Mongolia.

### Pathogen detection in rodents

Using the TBP Panel, 59 rodents from Jinghong City, Yunnan province, were screened. Internal controls achieved a 100% validity rate across all rodent liver samples, yielding a mean Cq of 28.9 ± 0.6. Adopting tiered Cq determination criteria, four bacterial pathogens were identified. The most prevalent was *Bartonella* spp. (67.8%), with 100% (5/5) detection in *Niviventer* and 63.0% (34/54) in *Rattus*. The remaining pathogens were found at lower prevalences, including *Borrelia* spp. (6.8%), *Rickettsia* spp. (5.1%), and *Ehrlichia* spp. (1.7%) (Table S13).

## Discussion

Diagnosing tick-borne diseases is a formidable challenge. Their clinical symptoms frequently overlap, and novel pathogens are continuously emerging [2, 29]. We developed and validated the TBP Panel, a multiplex qPCR system detecting 25 tick-borne pathogens across 8 multiplex assays. This study first established its utility in large-scale surveillance by characterizing the pathogen landscape in >2,300 ticks, and subsequently confirmed its clinical relevance by identifying etiologies in 7.9% of AFI patients. By standardizing data across vectors and patients, the panel effectively bridges epidemiological monitoring and clinical practice.

Pathogen selection was strictly guided by the China National Health Commission Catalogue of Pathogenic Microorganisms and recent literature, ensuring comprehensive coverage of clinical and biosafety threats [2, 19, 30]. The panel prioritizes highly prevalent agents (e.g. *A. phagocytophilum*, *Rickettsia*, SFTSV, and *B. microti*) to maximize diagnostic utility for most suspected TBD cases [2, 31, 32]. Furthermore, this study integrated emerging “orphan” pathogens, such as the recently discovered Wetland (WELV, 2024) and Xuecheng (XCV, 2025) viruses [30, 33], enabling continuous surveillance and early warning. Finally, serving as a tool for comprehensive surveillance, the Panel includes established zoonotic pathogens (e.g. NSDV and *Theileria* spp.) that impact both livestock and human health [34, 35]. However, the *Hepatozoon*/*Theileria* composite assay generated a late amplification signal (Cq > 35) for *Colpodella* sp. (GenBank: PZ278514) in one AFI patient. Notably, *Colpodella* is a rare tick-borne zoonotic pathogen associated with human febrile illness [36]. Sequence alignment revealed multiple mismatches with our assay, highlighting the need to establish stringent Cq cut-offs and incorporate dedicated *Colpodella* primers in future panel iterations.

The Panel's primary clinical value lies in resolving low-load infections. In both natural reservoirs (asymptomatic persistence) and early stage clinical patients (e.g. SFTSV or YEZV), pathogen loads frequently hover near conventional limits of detection (Cq ≥ 35) [11, 37]. This creates a “diagnostic gray zone” vulnerable to false negatives (stochastic Poisson effects) and false positives (late-cycle non-specific signals) [26]. Our large-scale screening directly quantified this challenge. Among all positive results, Cq values > 35 accounted for 21.4% (97/454) and 12.8% (6/47) of positive detections in ticks and rodents, respectively. Strikingly, this proportion of low-load infections reached 84.0% (21/25) in the AFI cohort. Therefore, relying on conventional, conservative cut-offs risks significant clinical omission.

To resolve these gray zones, we established a composite gold standard using amplicon sequencing, complemented by digital PCR (dPCR). By partitioning samples into thousands of independent micro-reactions, dPCR enables precise absolute quantification without standard curves and exhibits superior resilience against PCR inhibitors, making it suited for adjudicating low copy targets [38]. Because the high costs of dPCR (∼10× higher) preclude routine clinical use, the study deployed it for methodological validation. Based on this high-sensitivity adjudication, the Panel implemented a tiered diagnostic algorithm: a Cq ≤ 36.6 signifies a true positive (100% PPA with the gold standard), while Cq 36.6–39.6 defines an equivocal range requiring re-testing (bounded by the Cq 39.6 cut-off). This calibrated system successfully achieved 96.5% sensitivity and 96.5% specificity.

Deploying the TBP Panel for large-scale vector surveillance revealed a diverse, geographically focal pathogen landscape among 2,333 ticks (18.5% overall prevalence). To account for variable pool sizes and avoid the statistical underestimation inherent to simple pool positivity, we applied MLE to calculate the Estimated Infection Rate (EIR) per 100 ticks. This pooled-testing model is widely recognized and routinely employed in vector-borne disease surveillance to provide unbiased prevalence estimates across heterogeneous pool sizes [39]. These distribution patterns align robustly with established eco-epidemiological characteristics in China: Northeastern China is a hotspot for emerging viruses (ALSV, BJNV, and NOMV) [2, 40]. while *Ehrlichia* and *Borrelia* commonly detected in Central and Southern China, respectively [31, 41]. This regional variation is further elucidated by Zhao et al. [23], who underscored that the distribution of these specific pathogens is shaped by varying environmental factors and vector niche preferences.

A major strength of the TBP Panel is its dual-application utility, effectively bridging vector surveillance with clinical diagnostics. In the Qingdao AFI cohort, SFTSV and *Ehrlichia* spp. emerged as the most frequently detected pathogens. This pathogen spectrum aligns robustly with previous regional studies, which established *Haemaphysalis longicornis* as the predominant vector harboring both agents in the Qingdao region [42, 43]. Both pathogens pose severe public health threats: SFTSV is a highly endemic bunyavirus that triggers severe hemorrhagic fever with case fatality rates of 5–30% [8, 42], while human monocytic ehrlichiosis leads to hospitalization in up to 50% of patients and carries a 2–3% fatality rate if treatment is delayed [44]. Crucially, concurrent vector surveillance also detected SFTSV and *Ehrlichia* spp. in local tick populations.

However, an incomplete concordance exists between vector carriage and human infection profiles. For example, despite a high 7.4% carriage rate of *Borrelia* spp. in Qingdao ticks, no human case was detected, likely because our genus-level assay captured various non-human-pathogenic strains, which warrants further characterization and differentiation. Conversely, 0.9% of *Anaplasma phagocytophilum* were identified in AFI cohort while tick samples tested negative, a discrepancy potentially likely stemming from spatio-temporal sampling limitations. Furthermore, while the lack of reported bite history among AFI patients could not rule out the presence of unnoticed tick exposure, it may also indicate the possibility of atypical transmission routes, as pathogens like SFTSV can occasionally transmit via direct contact with infected blood or bodily fluids [45].

Conversely, the Inner Mongolia FUO cohort yielded only a single *Rickettsia*-positive case, despite the region being a well-documented natural focus characterized by diverse tick species [23, 46], abundant rodent reservoirs [47], and the established circulation of emerging viruses (e.g. ALSV and BJNV) [2]. Of note, the majority of this cohort were farmers and herdsmen (69.5%) with frequent animal contact (85.4%). Furthermore, qPCR-based screening detected the livestock-associated pathogen i.e. *Brucella* spp. in 15.5% cases [48], consistent with this occupational risk. Furthermore, limited by the available sample input, only 200 µL of blood from each FUO case were processed using a manual nucleic acid extraction kit, whereas 2 mL of whole blood from the AFI cohort were processed with a magnetic bead-based automated large-volume extraction system. Given the inherently low TBP loads in blood, this reduced volume likely compromised the detection yield in FUO cases, leading to underestimation of pathogen prevalence.

To validate the Panel in other biological matrices, it was tested on 59 rodent tissue samples from Jinghong City, Yunnan Province. The study detected a remarkably high prevalence of *Bartonella* spp. (67.8%), which aligns with the unique disease ecology of Southwestern China, a renowned biodiversity hotspot where diverse rodent populations (e.g. *Rattus tanezumi*) serve as primary natural reservoirs [49]. Notably, a previous screening of 443 AFI patients in neighboring Mengla County (Yunnan Province) did not detect any *Bartonella* infection [50]. Additionally, no matched tick samples from rodents were available for testing. However, given the limited clinical sample size and the geographical/temporal variance between sampling sites and timing, its potential as an underrecognized etiology of local human febrile illnesses cannot be ruled out.

While this study establishes a high throughput molecular detection platform, several limitations remain. First, 11 of the 25 target pathogens were not detected. Given our rigorous validation using both synthetic standards and genuine nucleic acid samples, this absence is likely attributable to limited sample sizes, constrained geographical distribution, and random sampling timeframes, rather than assay deficiencies. For example, WELV circulates primarily in summer (May–July) in Central China [51], whereas our samples from Henan and Jiangsu provinces were primarily collected in September. Furthermore, niche-specialized pathogens like TcTV-1 and TAMV in Xinjiang [46] may elude detection due to restricted local sampling (only 31 engorged ticks). Crucially, the lack of true-positive samples precluded the establishment of target-specific diagnostic sensitivity and appropriate ROC analysis for these low-prevalence agents; consequently, our overall pooled diagnostic performance metrics are primarily driven by targets with sufficient positive representations, such as *C. burnetii* and *Ehrlichia* spp. Nonetheless, retaining these emerging pathogens in routine surveillance remains essential to monitor spillover threats driven by expanding tick habitats and climate warming [52, 53]. Future studies will leverage Geographic Information Systems (GIS) to optimize sampling efficiency and capture low-prevalence agents [54].

Another analytical constraint is that sensitivity varied between sample types. The LOD for whole blood was superior to that of complex tick pools. This variance is likely attributable to the interplay of divergent extraction methodologies employed [55], and the concurrent matrix effects induced by endogenous inhibitors (e.g. chitin) within tick homogenates, which can actively interfere with PCR amplification [56, 57], Well-reflected by the MS2 external control [58]. Subsequent optimizations will focus on high efficiency extraction protocols and increased template loading to enhance sensitivity in arthropod samples. Furthermore, while the current study utilized the panel on screening ticks, wild rodents, and human clinical cases, the absence of livestock and domestic animal sampling limits our ability to fully characterize the human-animal-vector interface. Therefore, large-scale prospective clinical studies, integration with metagenomic data, and future inclusion of domestic animal surveillance are required to cement the long-term utility of this panel in advancing comprehensive multi-host surveillance.

## Conclusions

In summary, the TBP Panel serves as a validated, high-throughput tool that bridges large-scale surveillance with clinical diagnostics. By enabling rapid etiological identification of undifferentiated febrile illnesses, the Panel addresses a diagnostic gap in tick-borne disease management.

## Abbreviations

*A. phagocytophilum*, *Anaplasma phagocytophilum*; ALSV, Alongshan virus; Bacteriophage MS2, Emesvirus zinderi; BJNV, Beiji-nairovirus; *C*. *burnetii*, *Coxiella burnetii*; CCHFV, Crimean-Congo hemorrhagic fever virus; Cq: Quantification Cycle; CI: Confidence Interval; CV: Coefficient of Variation; E: Amplification Efficiency; *F*. *tularensis*, *Francisella tularensis*; JMTV, Jingmen tick virus; LOD: Limit of Detection; NOMV, Nuomin virus; NSDV, Nairobi sheep disease virus; *R*^2^: Coefficient of Determination; Rep: Repeatability; Repr: Reproducibility; SFTSV, Severe fever with thrombocytopenia syndrome virus; SGLV, Songling virus; TBDs, Tick-borne diseases; TBP, Tick-Borne Pathogen; *T*. *gondii*, *Toxoplasma gondii*; TBEV, Tick-borne encephalitis virus; TcTV-1, Tacheng tick virus-1; TAMV, Tamdy virus; WELV, Wetland virus; XCV, Xue-Cheng virus; YEZV, Yezo virus.

## Supplementary Material

Graphical Abstract.tif

Supplementary material.docx

## Data Availability

The datasets supporting the conclusions of this article are included within the article and its Supplementary materials.

## References

[1] de la Fuente J, Estrada-Pena A, Venzal JM, et al. Overview: ticks as vectors of pathogens that cause disease in humans and animals. Front Biosci. 2008;13:6938–6946. doi:10.2741/3200 18508706

[2] Wu YF, Zhang MZ, Su XL, et al. Emerging tick-borne diseases in mainland China over the past decade: a systematic review and mapping analysis. Lancet Reg Health West Pac. 2025;59:101599. doi:10.1016/j.lanwpc.2025.101599 40546707 PMC12182379

[3] Centers for Disease C, Prevention. Lyme disease surveillance and data 2023. https://www.cdc.gov/lyme/datasurveillance/index.html.

[4] Heglasová I, Víchová B, Kraljik J, et al. Molecular evidence and diversity of the spotted-fever group Rickettsia spp. in small mammals from natural, suburban and urban areas of Eastern Slovakia. Ticks Tick Borne Dis. 2018;9(6):1400–1406. doi:10.1016/j.ttbdis.2018.06.011 30207272

[5] Mannelli A, Bertolotti L, Gern L, et al. Ecology of Borrelia burgdorferi sensu lato in Europe: transmission dynamics in multi-host systems, influence of molecular processes and effects of climate change. FEMS Microbiol Rev. 2012;36(4):837–861. doi:10.1111/j.1574-6976.2011.00312.x 22091928

[6] de Souza WM, Weaver SC. Effects of climate change and human activities on vector-borne diseases. Nat Rev Microbiol. 2024;22(8):476–491. doi:10.1038/s41579-024-01026-0 38486116

[7] Rodino KG, Theel ES, Pritt BS. Update on North American tick-borne diseases and how to diagnose them. J Clin Microbiol. 2025;63(8):e0080723. doi:10.1128/jcm.00807-23 40643260 PMC12345162

[8] Sun H, Hu Q, Lu S, et al. Current status of severe fever with thrombocytopenia syndrome in China (Review). Int J Mol Med. 2025;56(5):169. doi:10.3892/ijmm.2025.5610 40849814 PMC12373438

[9] Paules CI, Marston HD, Bloom ME, et al. Tickborne diseases - confronting a growing threat. N Engl J Med. 2018;379(8):701–703. doi:10.1056/NEJMp1807870 30044925

[10] Kugeler KJ, Scotty E, Earley A, et al. Lyme disease testing practices, Wisconsin, USA, 2016-2019. Emerg Infect Dis. 2025;31(7):1421–1424. doi:10.3201/eid3107.250009 40562745 PMC12205465

[11] Ogata Y, Sato T, Kato K, et al. A case of tick-borne Yezo virus infection: concurrent detection in the patient and tick. Int J Infect Dis. 2024;143:107038. doi:10.1016/j.ijid.2024.107038 38580070

[12] Hillerdal H, Lager M, Grankvist A, et al. Are there other undiagnosed tick-borne infections in children being evaluated for Lyme neuroborreliosis? BMC Pediatr. 2026;26(1):565. doi:10.1186/s12887-026-07000-4 42298473 PMC13274043

[13] Xu S, Gui Z, Liu Z, et al. Development and evaluation of single-plex TaqMan real-time quantitative PCR assays for the detection of six tick-borne pathogenic viruses in northeastern China. Front Cell Infect Microbiol. 2026;16:1777543. doi:10.3389/fcimb.2026.1777543 42328165 PMC13275643

[14] Deurenberg RH, Bathoorn E, Chlebowicz MA, et al. Application of next generation sequencing in clinical microbiology and infection prevention. J Biotechnol. 2017;243:16–24. doi:10.1016/j.jbiotec.2016.12.022 28042011

[15] Rodino KG, Simner PJ. Status check: next-generation sequencing for infectious-disease diagnostics. J Clin Invest. 2024;134(4):e178003. doi:10.1172/jci178003 38357923 PMC10866643

[16] Holmgaard DB, Barnadas C, Mirbarati SH, et al. Detection and identification of Acanthamoeba and other nonviral causes of infectious keratitis in corneal scrapings by real-time PCR and next-generation sequencing-based 16S-18S gene analysis. J Clin Microbiol. 2021;59(2):e02224-20. doi:10.1128/jcm.02224-20 33239372 PMC8111161

[17] Grundy BS, Parikh H, Jacob S, et al. Pathogen detection using metagenomic next-generation sequencing of plasma samples from patients with sepsis in Uganda. Microbiol Spectr. 2023;11(1):e0431222. doi:10.1128/spectrum.04312-22 36625651 PMC9927450

[18] Ni XB, Cui XM, Liu JY, et al. Metavirome of 31 tick species provides a compendium of 1,801 RNA virus genomes. Nat Microbiol. 2023;8(1):162–173. doi:10.1038/s41564-022-01275-w 36604510 PMC9816062

[19] Jia N, Wang J, Shi W, et al. Large-scale comparative analyses of tick genomes elucidate their genetic diversity and vector capacities. Cell. 2020;182(5):1328–40.e13. doi:10.1016/j.cell.2020.07.023 32814014

[20] National Pathogen Resource Center. Infectious disease database 2026. https://www.nprc.org.cn/.

[21] L J, W S, Z Y. Assessment of four DNA fragments (COI, 16S rDNA, ITS2, 12S rDNA) for species identification of the Ixodida (Acari: Ixodida). Parasit Vectors. 2014;7:93. doi:10.1186/1756-3305-7-93. 24589289 PMC3945964

[22] M AJ, B MD, M-C. S. Mitochondrial 16S rDNA sequences and phylogenetic relationships of species of Rhipicephalus and other tick genera among Metastriata (Acari: Ixodidae). Parasitol Res. 1998;84(6):478–484. doi:10.1007/s004360050433 9660138

[23] Zhao GP, Wang YX, Fan ZW, et al. Mapping ticks and tick-borne pathogens in China. Nat Commun. 2021;12(1):1075. doi:10.1038/s41467-021-21375-1 33597544 PMC7889899

[24] Breslauer KJ, Frank R, Blöcker H, et al. Predicting DNA duplex stability from the base sequence. Proc Natl Acad Sci USA. 1986;83(11):3746–3750. doi:10.1073/pnas.83.11.3746 3459152 PMC323600

[25] Kodani M, Winchell JM. Engineered combined-positive-control template for real-time reverse transcription-PCR in multiple-pathogen-detection assays. J Clin Microbiol. 2012;50(3):1057–1060. doi:10.1128/jcm.05987-11 22170926 PMC3295119

[26] Bustin SA, Ruijter JM, van den Hoff MJB, et al. MIQE 2.0: revision of the minimum information for publication of quantitative real-time PCR experiments guidelines. Clin Chem. 2025: 634–651. doi: 10.1093/clinchem/hvaf043 40272429

[27] Centers for Disease C, Prevention. Mosquito surveillance software 2025. https://www.cdc.gov/mosquitoes/php/toolkit/mosquito-surveillance-software.html.

[28] Jiang S, Li H, Zhang L, et al. Generic diagramming platform (GDP): a comprehensive database of high-quality biomedical graphics. Nucleic Acids Res. 2025;53(D1):D1670–D16d6. doi:10.1093/nar/gkae973 39470721 PMC11701665

[29] Fang LQ, Liu K, Li XL, et al. Emerging tick-borne infections in mainland China: an increasing public health threat. Lancet Infect Dis. 2015;15(12):1467–1479. doi:10.1016/s1473-3099(15)00177-2 26453241 PMC4870934

[30] Zhang XA, Ma YD, Zhang YF, et al. A New orthonairovirus associated with human febrile illness. N Engl J Med. 2024;391(9):821–831. doi:10.1056/NEJMoa2313722 39231344

[31] Huang XB, Tang T, Chen JJ, et al. The global distribution and risk prediction of Anaplasmataceae species: a systematic review and geospatial modelling analysis. EBioMedicine. 2025;115:105722. doi:10.1016/j.ebiom.2025.105722 40273471 PMC12051633

[32] Kumar A, O'Bryan J, Krause PJ. The global emergence of human Babesiosis. Pathogens. 2021;10(11):1447. doi:10.3390/pathogens10111447 34832603 PMC8623124

[33] Zhang MZ, Bian C, Ye RZ, et al. Human infection with a novel tickborne orthonairovirus species in China. N Engl J Med. 2025;392(2):200–202. doi:10.1056/NEJMc2410853 39778175

[34] Yao XY, Wang MH, Zhang XL, et al. Epidemiological, virological, and pathogenic insights into Nairobi sheep disease virus infection in sheep and goats in China. J Virol. 2025;99(6):e0000625. doi:10.1128/jvi.00006-25 40396758 PMC12172417

[35] Xiang R, Du CH, Zhao YL, Luo Z, Li M, Zeng DN, et al. Theileria luwenshuni and Novel Babesia spp. Infections in humans, Yunnan Province, China. Emerg Infect Dis. 2025;31(9):1764–1773. doi:10.3201/eid3109.241919 40867024 PMC12407207

[36] Zhao Y, Cao Z, Li S, et al. Biological characteristics and epidemiological insights into the zoonotic potential of Colpodella spp.: a scoping review. Infect Dis Poverty. 2025;14(1):91. doi:10.1186/s40249-025-01361-1 40877989 PMC12392618

[37] Yang ZD, Hu JG, Lu QB, et al. The prospective evaluation of viral loads in patients with severe fever with thrombocytopenia syndrome. J Clin Virol. 2016;78:123–128. doi:10.1016/j.jcv.2016.03.017 27062673

[38] Huggett JF. The digital MIQE guidelines update: minimum information for publication of quantitative digital PCR experiments for 2020. Clin Chem. 2020;66(8):1012–1029. doi:10.1093/clinchem/hvaa125 32746458

[39] Fang Y, Li XS, Zhang W, et al. Molecular epidemiology of mosquito-borne viruses at the China-Myanmar border: discovery of a potential epidemic focus of Japanese encephalitis. Infect Dis Poverty. 2021;10(1):57. doi:10.1186/s40249-021-00838-z 33902684 PMC8073957

[40] Guo M, Liu H, Zhou H, et al. The risk of pathogenic tick-borne viruses in Northeast Asia: a genomic and ecological modelling study. Sci China Life Sci. 2026;69(6):2060–2072. doi:10.1007/s11427-025-3146-2 41706271

[41] Zhang XA, Tian F, Li Y, et al. Molecular detection and identification of relapsing fever Borrelia in ticks and wild small mammals in China. Emerg Microbes Infect. 2022;11(1):2632–2635. doi:10.1080/22221751.2022.2134054 36214427 PMC9639508

[42] Leng Y, Mu HZ, Cui N, et al. Molecular evolution and spatial transmission of severe fever with thrombocytopenia syndrome virus. Nat Commun. 2026;17(1):4499. doi:10.1038/s41467-026-71008-8 41896574 PMC13187474

[43] Luo L, Sun J, Yan J, et al. Detection of a novel Ehrlichia species in Haemaphysalis longicornis tick from China. Vector Borne Zoonotic Dis. 2016;16(6):363–367. doi:10.1089/vbz.2015.1898 27135624 PMC4884337

[44] Center CUIM. Ehrlichiosis 2025. https://www.columbia-lyme.org/ehrlichiosis.

[45] Zheng W, Zhou H, Carr MJ, et al. Epidemic potential of SFTSV: increasing evidence for non-vector-borne transmission. Lancet Microbe. 2026;7(2):101271. doi:10.1016/j.lanmic.2025.101271 41205620

[46] Zhou H, Liu H, Wang YX, et al. The risk of human- and mammal-infecting tick-borne viruses in Northwest China and adjacent countries. Nat Commun. 2025;17(1):175. doi:10.1038/s41467-025-66873-8 41330909 PMC12780119

[47] Su S, Cui MY, Xing LL, et al. Metatranscriptomic analysis reveals the diversity of RNA viruses in ticks in Inner Mongolia, China. PLoS Negl Trop Dis. 2024;18(12):e0012706. doi:10.1371/journal.pntd.0012706 39661583 PMC11634002

[48] Zhang T, Li J, Hai Y, et al. Prevalence and detection of Brucella infection in people with fever of unknown origin in Inner Mongolia, China. Front Cell Infect Microbiol. 2026;16:1838461. doi:10.3389/fcimb.2026.1838461 42389508 PMC13318869

[49] Luo YY, Yu D, Zhang HZ, et al. Molecular detection of Bartonella species in wild small mammals in western Yunnan Province, China. Front Vet Sci. 2023;10:1301316. doi:10.3389/fvets.2023.1301316 38076558 PMC10703294

[50] Rainey JJ, Siesel C, Guo X, et al. Etiology of acute febrile illnesses in Southern China: findings from a two-year sentinel surveillance project, 2017-2019. PLoS One. 2022;17(6):e0270586. doi:10.1371/journal.pone.0270586 35763515 PMC9239456

[51] Yuan HX, Lv XL, Zhang MQ, et al. Genetic diversity and expanded epidemic area of novel tick-borne pathogen wetland virus in ticks, wild and domestic animals, and patient in China. Emerg Microbes Infect. 2025;14(1):2502003. doi:10.1080/22221751.2025.2502003 40314229 PMC12100960

[52] Ni XB, Pei Y, Ye YT, et al. Ecoclimate drivers shape virome diversity in a globally invasive tick species. ISME J. 2024;18(1):wrae087. doi:10.1093/ismejo/wrae087 38747389 PMC11187987

[53] Pei T, Nwanade CF, Wang Z, et al. Adaptive restructuring of the microbiota promotes overwintering survival of the tick Dermacentor silvarum. Parasit Vectors. 2026;19(1):318. doi:10.1186/s13071-026-07457-3 42231482 PMC13445671

[54] Eisen L, Eisen RJ. Using geographic information systems and decision support systems for the prediction, prevention, and control of vector-borne diseases. Annu Rev Entomol. 2011;56:41–61. doi:10.1146/annurev-ento-120709-144847 20868280

[55] Made Artika I, Ma'roef CN, Johar E, et al. Comprehensive review on viral RNA extraction strategies for enhanced molecular diagnostics. Interdiscip Perspect Infect Dis. 2025;2025:5579320, doi:10.1155/ipid/5579320 41477104 PMC12752832

[56] Reifenberger GC, Thomas BA, Rhodes DVL. Comparison of DNA extraction and amplification techniques for use with engorged hard-bodied ticks. Microorganisms. 2022;10(6):1254. doi:10.3390/microorganisms10061254 35744772 PMC9228219

[57] Okeyo M, Hartberger C, Margos G, et al. Comparison of methods for economic and efficient tick and Borrelia DNA purification. Ticks Tick Borne Dis. 2019;10(5):1041–1045. doi:10.1016/j.ttbdis.2019.05.002 31171466

[58] Liu J, Ochieng C, Wiersma S, et al. Development of a TaqMan array card for acute-febrile-illness outbreak investigation and surveillance of emerging pathogens, including Ebola virus. J Clin Microbiol. 2016;54(1):49–58. doi:10.1128/jcm.02257-15 26491176 PMC4702733

