# Supplementary material for: Establishment and application of a multiplex qPCR panel for multi-host surveillance of tick-Borne pathogens
Source: Emerg Microbes Infect. 2026 Sep 12;15(1):2731498. doi: 10.1080/22221751.2026.2731498 (PMC13573549; doi:10.1080/22221751.2026.2731498)
Supplement: Supplementary file 1 [file TEMI_A_2731498_SM4849.docx]

**Supplementary Information**

Table of contents

Supplementary Table S1…………………………..………………...……….….....2

Supplementary Table S2…………………………..………………...……….…….6

Supplementary Table S3…………………………..….…………...…………….…7

Supplementary Table S4………………………………………………...…...….....9

Supplementary Table S5………………………………………………...…...…...15

Supplementary Table S6………………………………………………...….....….17

Supplementary Table S7……………………………………………………..…...20

Supplementary Table S8……………………………………………………..…...25

Supplementary Table S9……………………………………………………..…...32

Supplementary Table S10……………………………………………..……..…...35

Supplementary Table S11……………………………………………..……..…...38

Supplementary Table S12……………………………………………..……..…...40

Supplementary Table S13……………………………………………..……..…...42

Supplementary Figure S1………………………………………………………...43

Supplementary Table S1. Positive rate of pathogens in ticks from five eco-climate regions in China^*^

| Eco-climate regions (NTP / NTT) | Province | Tick species (NTP / NTT) | *A. phagocytophilum* | *Bartonella* spp. | *Borrelia* spp. | *C*. *burnetii* | *Ehrlichia* spp. | *Rickettsia* spp. |
| --- | --- | --- | --- | --- | --- | --- | --- | --- |
| Northeastern China (9 / 105) | Jilin | *Haemaphysalis concinna* (1/8) | 0 | 0 | 1 | 0 | 0 | 1 |
|  |  | *Haemaphysalis hystricis* (1/10) | 0 | 0 | 1 | 0 | 1 | 1 |
|  |  | *Ixodes persulcatus* (2/33) | 0 | 1 | 2 | 0 | 1 | 2 |
|  | Inner-Mongolia | *Ixodes persulcatus* (2/23) | 0 | 0 | 2 | 0 | 2 | 2 |
|  |  | *Ixodes crenulatus* (2/21) | 0 | 0 | 2 | 0 | 1 | 1 |
|  | Liaoning | *Haemaphysalis longicornis* (1/10) | 0 | 0 | 1 | 0 | 0 | 1 |
|  | NPP, Estimated tick infection rate (%, 95% CI) | | 0, 0 (0.0 - 3.5) | 1, 1 (0.1 - 4.5) | 9, 8.6 (4.3 - 15.1) | 0, 0 (0.0 - 3.5) | 5, 4.8 (1.8 - 10.2) | 8, 7.6 (3.6 - 13.9) |
| Northern China  (143 / 1633) | Henan | *Haemaphysalis longicornis* (2/24) | 0 | 0 | 1 | 0 | 1 | 2 |
|  |  | *Rhipicephalus microplus* (1/16) | 0 | 0 | 1 | 0 | 1 | 0 |
|  | Jiangsu | *Haemaphysalis longicornis* (3/3) | 0 | 0 | 1 | 0 | 2 | 1 |
|  |  | *Haemaphysalis flava* (1/74) | 0 | 0 | 1 | 0 | 0 | 1 |
|  | Shandong | *Haemaphysalis longicornis* (131/1390) | 0 | 15 | 64 | 9 | 33 | 17 |
|  |  | *Rhipicephalus microplus* (7/14) | 0 | 0 | 5 | 0 | 0 | 0 |
|  |  | *Haemaphysalis flava* (1/78) | 0 | 0 | 1 | 0 | 0 | 1 |
|  | Shanxi | *Haemaphysalis longicornis* (2/22) | 0 | 0 | 2 | 0 | 1 | 1 |
|  | Shannxi | *Haemaphysalis longicornis* (1/10) | 0 | 0 | 1 | 0 | 0 | 1 |
|  |  | *Rhipicephalus microplus* (1/2) | 0 | 0 | 1 | 0 | 1 | 1 |
|  | NPP, Estimated tick infection rate (%, 95% CI) | | 0, 0 (0.0 - 0.2) | 15, 1 (0.6 - 1.5) | 78, 7.4 (6.0 - 9.0) | 9, 0.6 (0.3 - 1.0) | 39, 2.7 (2.0 - 3.6) | 25, 1.8 (1.2 - 2.6) |
| Inner Mongolia-Xinjiang  (27 / 298) | Gansu | *Hyalomma asiaticum* (5/190) | 0 | 0 | 0 | 0 | 1 | 0 |
|  | Xinjiang | *Dermacentor nuttalli* (5/31) | 0 | 0 | 5 | 4 | 1 | 5 |
|  |  | *Hyalomma asiaticum* (11/48) | 1 | 0 | 11 | 10 | 2 | 4 |
|  |  | *Hyalomma excavatum* (6/29) | 2 | 0 | 6 | 5 | 1 | 4 |
|  | NPP, Estimated tick infection rate (%, 95% CI) | | 3, 1 (0.3 - 2.5) | 0, 0 (0.0 - 1.1) | 22, 8.5 (6.7 - 11.3) | 19, 6.8 (5.3 - 9.3) | 5, 1.6 (0.7 - 3.4) | 13, 4.6 (3.2 - 6.9) |
| Central China  (14 / 144) | Anhui | *Haemaphysalis longicornis* (2/5) | 0 | 0 | 1 | 0 | 2 | 1 |
|  | Chongqing | *Haemaphysalis hystricis* (1/2) | 0 | 0 | 1 | 0 | 0 | 1 |
|  | Fujian | *Rhipicephalus sanguineus* (1/3) | 0 | 0 | 1 | 0 | 0 | 0 |
|  | Guizhou | *Haemaphysalis longicornis* (1/10) | 0 | 0 | 1 | 0 | 1 | 0 |
|  | Hunan | *Rhipicephalus microplus* (1/3) | 0 | 0 | 1 | 0 | 0 | 1 |
|  | Zhejiang | *Rhipicephalus sanguineus* (8/121) | 0 | 1 | 3 | 0 | 8 | 0 |
|  | NPP, Estimated tick infection rate (%, 95% CI) | | 0, 0 (0.0 - 0.3) | 1, 0.7 (0.04 - 3.4) | 8, 6.8 (3.6 - 12.7) | 0, 0 (0.0 - 0.3) | 11, 19.3 (9.9 - 47.1) | 3, 2.0 ( 0.6 - 5.3) |
| Southern China  (32 / 153) | Guangdong | *Rhipicephalus sanguineus* (3/41) | 0 | 1 | 3 | 0 | 0 | 1 |
|  | Guangxi | *Rhipicephalus sanguineus* (12/48) | 0 | 0 | 2 | 0 | 1 | 1 |
|  |  | *Ixodes granulatus* (17/64) | 0 | 1 | 12 | 0 | 2 | 5 |
|  | NPP, Overall Prevalence (%, 95% CI) | | 0, 0 (0 - 2.3) | 2, 1.3 (0.2 - 4.3) | 17, 17.2 (10.7 - 26.7) | 0, 0 (0 - 2.3) | 3, 2.1 (0.6 - 5.5) | 7, 5.0 (2.3 - 9.5) |
| Total (NTP/NTT = 225 / 2333): NPP, Estimated individual infection rate (%, 95% CI) | | | 3, 0.1 (0.03 - 0.3) | 19, 0.8 (0.5 - 1.3) | 134, 8.6 (7.6 - 10.0) | 28, 1.2 (0.9-1.7) | 63, 3.1 (2.5 - 3.9) | 56, 2.8 (2.2 - 3.6) |

| Eco-climate regions (NTP/NTT) | Province | Tick species (NTP/NTT) | ALSV | BJNV | NSDV | NOMV | SFTSV |
| --- | --- | --- | --- | --- | --- | --- | --- |
| Northeastern China (9/105) | Jilin | *Haemaphysalis concinna* (1/8) | 0 | 0 | 0 | 0 | 0 |
|  |  | *Haemaphysalis hystricis* (1/10) | 0 | 1 | 0 | 1 | 0 |
|  |  | *Ixodes persulcatus* (2/33) | 0 | 2 | 0 | 1 | 0 |
|  | Inner-Mongolia | *Ixodes persulcatus* (2/23) | 1 | 2 | 0 | 2 | 0 |
|  |  | *Ixodes crenulatus* (2/21) | 0 | 1 | 0 | 1 | 0 |
|  | Liaoning | *Haemaphysalis longicornis* (1/10) | 0 | 0 | 0 | 0 | 0 |
|  | NPP, Estimated tick infection rate (%, 95% CI) | | 1, 1 (0.1 - 4.5) | 5, 4.8 (1.8 - 10.2) | 0, 0 (0.0 - 3.5) | 5, 6.4 (2.5 -15.0) | 0, 0 (0.0 - 3.5) |
| Northern China  (143/1633) | Henan | *Haemaphysalis longicornis* (2/24) | 0 | 0 | 0 | 0 | 0 |
|  |  | *Rhipicephalus microplus* (1/16) | 0 | 0 | 0 | 0 | 0 |
|  | Jiangsu | *Haemaphysalis longicornis* (3/3) | 0 | 0 | 0 | 0 | 0 |
|  |  | *Haemaphysalis flava* (1/74) | 0 | 0 | 0 | 0 | 0 |
|  | Shandong | *Haemaphysalis longicornis* (131/1390) | 0 | 0 | 6 | 0 | 5 |
|  |  | *Rhipicephalus microplus* (7/14) | 0 | 0 | 0 | 0 | 0 |
|  |  | *Haemaphysalis flava* (1/78) | 0 | 0 | 0 | 0 | 0 |
|  | Shanxi | *Haemaphysalis longicornis* (2/22) | 0 | 0 | 0 | 0 | 0 |
|  | Shannxi | *Haemaphysalis longicornis* (1/10) | 0 | 0 | 0 | 0 | 0 |
|  |  | *Rhipicephalus microplus* (1/2) | 0 | 0 | 0 | 0 | 0 |
|  | NPP, Estimated tick infection rate (%, 95% CI) | | 0, 0 (0.0 - 0.2) | 0, 0 (0.0 - 0.2) | 6, 0.4 (0.2 - 0.8) | 0, 0 (0.0 - 0.2) | 5, 0.3 (0.1 - 0.7) |
| Inner Mongolia-Xinjiang  (27/298) | Gansu | *Hyalomma asiaticum* (5/190) | 0 | 0 | 0 | 0 | 0 |
|  | Xinjiang | *Dermacentor nuttalli* (5/31) | 0 | 0 | 0 | 0 | 0 |
|  |  | *Hyalomma asiaticum* (11/48) | 0 | 0 | 0 | 0 | 0 |
|  |  | *Hyalomma excavatum* (6/29) | 0 | 0 | 0 | 0 | 0 |
|  | NPP, Estimated tick infection rate (%, 95% CI) | | 0, 0 (0.0 - 1.1) | 0, 0 (0 - 1.1) | 0, 0 (0 - 1.1) | 0, 0 (0 - 1.1) | 0, 0 (0 - 1.1) |
| Central China (14/144) | Anhui | *Haemaphysalis longicornis* (2/5) | 0 | 0 | 0 | 0 | 0 |
|  | Chongqing | *Haemaphysalis hystricis* (1/2) | 0 | 0 | 0 | 0 | 0 |
|  | Fujian | *Rhipicephalus sanguineus* (1/3) | 0 | 0 | 0 | 0 | 0 |
|  | Guizhou | *Haemaphysalis longicornis* (1/10) | 0 | 0 | 0 | 0 | 0 |
|  | Hunan | *Rhipicephalus microplus* (1/3) | 0 | 0 | 0 | 0 | 0 |
|  | Zhejiang | *Rhipicephalus sanguineus* (8/121) | 0 | 0 | 0 | 0 | 2 |
|  | NPP, Estimated tick al infection rate (%, 95% CI) | | 0, 0 (0.0 - 0.3) | 0, 0 (0.0 - 0.3) | 0, 0 (0.0 - 0.3) | 0, 0 (0.0 - 0.3) | 2, 1.5 (0.3 - 4.9) |
| Southern China  (32/153) | Guangdong | *Rhipicephalus sanguineus* (3/41) | 0 | 0 | 0 | 0 | 0 |
|  | Guangxi | *Rhipicephalus sanguineus* (12/48) | 0 | 0 | 0 | 0 | 0 |
|  |  | *Ixodes granulatus* (17/64) | 0 | 0 | 0 | 0 | 0 |
|  | NPP, Estimated tick infection rate (%, 95% CI) | | 0, 0 (0 - 2.3) | 0, 0 (0 - 2.2) | 0, 0 (0 - 2.3) | 0, 0 (0 - 2.3) | 0, 0 (0 - 2.3) |
| Total (NTP/NTT = 225/2333): NPP, Estimated individual infection rate (%, 95% CI) | | | 1, 0 (0.0 - 0.2) | 6, 0.3 (0.1 - 0.5) | 6, 0.3 (0.1 - 0.5) | 5, 0.2 (0.1 - 0.5) | 7, 0.3 (0.1 - 0.6) |
| Eco-climate regions (NTP/NTT) | Province | Tick species | *Babesia* spp. | | *Hepatozoon* spp*.* / *Theileria* spp*.* | | |
| Northeastern China  (9/105) | Jilin | *Haemaphysalis concinna* (1/8) | 1 | | 0 | | |
|  |  | *Haemaphysalis hystricis* (1/10) | 1 | | 0 | | |
|  |  | *Ixodes persulcatus* (2/33) | 1 | | 1 | | |
|  | Inner Mongolia | *Ixodes persulcatus* (2/23) | 2 | | 1 | | |
|  |  | *Ixodes crenulatus* (2/21) | 0 | | 0 | | |
|  | Liaoning | *Haemaphysalis longicornis* (1/10) | 0 | | 0 | | |
|  | NPP, Estimated tick infection rate (%, 95% CI) | | 5, 4.8 (1.8 - 10.2) | | 2, 1.9 (0.3 - 6.1） | | |
| Northern China  (143/1633) | Henan | *Haemaphysalis longicornis* (2/24) | 0 | | 1 | | |
|  |  | *Rhipicephalus microplus* (1/16) | 0 | | 1 | | |
|  | Jiangsu | *Haemaphysalis longicornis* (3/3) | 1 | | 1 | | |
|  |  | *Haemaphysalis flava* (1/74) | 1 | | 0 | | |
|  | Shandong | *Haemaphysalis longicornis* (131/1390) | 8 | | 71 | | |
|  |  | *Rhipicephalus microplus* (7/14) | 5 | | 7 | | |
|  |  | *Haemaphysalis flava* (1/78) | 0 | | 1 | | |
|  | Shanxi | *Haemaphysalis longicornis* (2/22) | 0 | | 1 | | |
|  | Shannxi | *Haemaphysalis longicornis* (1/10) | 0 | | 1 | | |
|  |  | *Rhipicephalus microplus* (1/2) | 1 | | 1 | | |
|  | NPP, Estimated tick infection rate (%, 95% CI) | | 16, 1 (0.6 - 1.6) | | 85, 8.4 (6.9 - 10.1)  ) | | |
| Inner Mongolia-Xinjiang  (27/298) | Gansu | *Hyalomma asiaticum* (5/190) | 0 | | 0 | | |
|  | Xinjiang | *Dermacentor nuttalli* (5/31) | 0 | | 1 | | |
|  |  | *Hyalomma asiaticum* (11/48) | 1 | | 0 | | |
|  |  | *Hyalomma excavatum* (6/29) | 0 | | 3 | | |
|  | NPP, Estimated tick infection rate (%, 95% CI) | | 1, 0.3 (0.02 - 1.5) | | 4, 1.3 (0.5 - 3.0) | | |
| Central China (14/144) | Anhui | *Haemaphysalis longicornis* (2/5) | 0 | | 1 | | |
|  | Chongqing | *Haemaphysalis hystricis* (1/2) | 0 | | 0 | | |
|  | Fujian | *Rhipicephalus sanguineus* (1/3) | 0 | | 0 | | |
|  | Guizhou | *Haemaphysalis longicornis* (1/10) | 0 | | 1 | | |
|  | Hunan | *Rhipicephalus microplus* (1/3) | 0 | | 1 | | |
|  | Zhejiang | *Rhipicephalus sanguineus* (8/121) | 1 | | 3 | | |
|  | NPP, Estimated tick infection rate (% , 95% CI) | | 1, 0.7 (0.04 - 3.4) | | 6, 5.0 (2.2 - 10.3) | | |
| Southern China  (32/153) | Guangdong | *Rhipicephalus sanguineus* (3/41) | 0  5 | | 0 | | |
|  | Guangxi | *Rhipicephalus sanguineus* (12/48) | 5 | | 0 | | |
|  |  | *Ixodes granulatus* (17/64) | 0 | | 1 | | |
|  | NPP, Estimated tick infection rate (%, 95% CI) | | 5, 3.3 (1.3 - 7.1) | | 1, 0.7 (0.03 - 3.1) | | |
| Total (NTP/NTT = 225/2333) : NPP, Estimated individual infection rate (%, 95% CI) | | | 28, 1.3 (0.9 - 1.8) | | 98, 5.8 (4.8 - 6.9) | | |

**Note**: ^*^Estimated individual infection rate (%, 95% CI): The values represent the Maximum Likelihood Estimation (MLE) of the infection rate per 100 individual ticks, along with their 95% confidence intervals. MLE values were calculated using the PooledInfRate software (developed by the US CDC) to accurately estimate the pathogen prevalence by individual tick based on the detection rate of pooled samples, accounting for pool sizes and the number of positive pools.

**Abbreviations**: NPP, No. of positive pools; NTP, No. of tick pools; NTT, No, of tested ticks.

Supplementary Table S2. Primer sequences for identifying tick species

| Primer | Target | Sequence (5′ - 3′) | Sizes (bp) | T_a_ (℃) | Reference |
| --- | --- | --- | --- | --- | --- |
| Ixodids-tick-16S | mitochondrial 16S rRNA | F: CCGGTCTGAACTCAGATCAAGT | 460 | 48 | (1) |
|  |  | R: GCTCAATGATTTTTTAAATTGCTGT |  |  |  |
| Ixodids-tick-COI | cytochrome oxidase I | F: GGAACAATATATTTAATTTTTGG | 820 | 55 | (2) |
|  |  | R: ATCTATCCCTACTGTAAATATATG |  |  |  |

**Abbreviations**: T_a,_ Annealing temperature; F, forward primer; R, reverse primer.

**Reference**

1.	AJ M, MD B, S M-C. Mitochondrial 16S rDNA sequences and phylogenetic relationships of species of Rhipicephalus and other tick genera among Metastriata (Acari: Ixodidae). Parasitol Res. 1998;84(6):478-84. https://doi.org/10.1007/s004360050433

2.	J L, S W, Y Z. Assessment of four DNA fragments (COI, 16S rDNA, ITS2, 12S rDNA) for species identification of the Ixodida (Acari: Ixodida). Parasit Vectors. 2014;7:93. https://doi.org/10.1186/1756-3305-7-93

Supplementary Table S3. Baseline characteristics of acute fever illness (AFI) patients in Qingdao and fever of unknown origin (FUO) patients in Inner Mongolia

| Characteristic | AFI patients^a^ (n = 317) | FUO patients^b^ (n = 236) |
| --- | --- | --- |
| Age, years, median (IQR) | 46 (31 - 66) | 55 (48 - 62) |
| Body temperature, °C, median (IQR) | 38.7 (38.0 - 39.2) | ND |
| Duration of fever, days, median (IQR) | 2 (1 - 4) | ND |
| *Gender, n (%)* | | |
| Female | 177 (55.8) | 141 (59.8) |
| Male | 140 (44.2) | 95 (40.3) |
| *Antibiotic use* | | |
| Yes | 51 (16.1) | 70 (29.7) |
| No | 266 (83.9) | 166 (70.3) |
| *Clinical symptoms, n (%)* | | |
| Systemic and musculoskeletal pain | 40 (12.6) | 225 (95.3) |
| Respiratory symptoms | 112 (35.3) | 0 (0.0) |
| CNS Manifestations | 71 (22.1) | 0 (0.0) |
| Gastrointestinal symptoms | 31 (9.8) | 12 (5.1) |

**Abbreviations**: IQR, Interquartile Range; ND, Not Determined.

**Note**: ^a.^Qingdao AFI cohort inclusion criteria: (i) Axillary temperature ≥ 38°C within 14 days of onset, presenting without definitive signs of a localized gastrointestinal infection (defined as ≥ 3 loose stools within 24 hours) or respiratory tract infection (e.g., productive cough, pulmonary rales, or inflammatory lesions on chest X-ray); (ii) Availability of complete clinical records, including medical history and routine blood results. Exclusion Criteria: (i) Fever attributable to known non-infectious causes (e.g., autoimmune diseases, drug fever, metabolic disorders, trauma); (ii) Pre-existing immunodeficiency (e.g., acquired immune deficiency syndrome); (iii) Active malignancy; (iv) Pregnancy; (v) A definitive diagnosis of another infectious disease (e.g., urinary tract infection, cellulitis).; ^b.^Inner Mongolia FUO cohort inclusion criteria: (i) Patients presented with fever of unknown origin, characterized by high fever and non-specific symptoms such as headache and myalgia, without a clear localizing source of infection upon initial clinical evaluation; (ii) Completion of a standardized questionnaire collecting demographic data (age, sex, occupation, residence), clinical symptoms.

Supplementary Table S4. Primer and probe sequences for the TBP Panel

| Assay | Pathogen | Target | Sequence (5′ - 3′) | Concentration  (nM) | Size  (bp) | Reference |
| --- | --- | --- | --- | --- | --- | --- |
| 1 | BJNV^a^ | S | F: GAGTCGGAACATCTTTTTCACC | 700 | 102 | This study |
|  |  |  | R: TGGGACAACCTCAACATTCA | 700 |  |  |
|  |  |  | P: Cy5-CCCTTCCARAACACTCACAACCTGTC-BHQ2 | 200 |  |  |
|  | JMTV^a^ | Segment 4 | F: TCGTGAACCTGTACTACTACYTG | 800 | 128 | (1) |
|  |  |  | R: CATTARRAGTGGGTAACCGT | 800 |  |  |
|  |  |  | P: FAM-ACCATCACAAACGTGCCTGCG-BHQ1 | 250 |  |  |
|  | XCV^a^ | S | F: AAGTGGAGCCAGTCCTATGAT | 800 | 150 | This study |
|  |  |  | R: CTTCACCACCGARTCCTG | 800 |  |  |
|  |  |  | P: VIC-AGATCAACCAGTACACCAAGGC-MGB | 250 |  |  |
| 2 | *Bartonella* spp.^b^ | *ssrA* | F: GGCTAAATNAGTAGTTGCAAAYGACA | 400 | 133 | (2) |
|  |  |  | R: GCTTCTGTTGCCAGGTG | 400 |  |  |
|  |  |  | P: FAM-ACCCCGCTTAAACCTGCGACG-MGB | 250 |  |  |
|  | *Borrelia* spp.^b^ | 16S rRNA | F: AGCYTTTAAAGCTTCGCTTGTAG | 400 | 148 | (3) |
|  |  |  | R: GCCTCCCGTAGGAGTCTGG | 400 |  |  |
|  |  |  | P: Cy5-CCGGCCTGAGAGGGTGAWCGG-BHQ2 | 250 |  |  |
|  | TBEV^a^ | NS5 | F: CGTACAGGTCCTGGCAGTA | 400 | 195 | (4) |
|  |  |  | R: GAGCCTTGGTGTCAACCTT | 400 |  |  |
|  |  |  | P: ROX-TTTGAACACTCGCTGCTGTCCAAAG-BHQ2 | 250 |  |  |
|  | YEZV^a^ | L | F: CTCAACCTGCTTCCAACCTATC | 500 | 108 | (5) |
|  |  |  | R: GGTGTAAAGCCCAACATCCT | 500 |  |  |
|  |  |  | P: VIC-CCAAGGAAGCACACAGATGGGTACA-BHQ1 | 250 |  |  |
| 3 | ALSV^a^ | NS3 | F: CATCGTRCTAAGCAGGAA | 400 | 128 | (6) |
|  |  |  | R: CACAGCATCCAGGTCATA | 400 |  |  |
|  |  |  | P: VIC-TGTTCAARCAGTACCCGA-MGB | 250 |  |  |
|  | *F. tularensis*^b^ | *tul4* | F: CARCATACAATAATAACCCACAAG | 400 | 124 | This study |
|  |  |  | R: GCCCAAGTTTTATCGTTCTKC | 400 |  |  |
|  |  |  | P: Cy5-TTCTAAGTGCCATGATACAAGCT-BHQ2 | 250 |  |  |
|  | SFTSV | L | F: AGTCTAGGTCATCTGATCCGTTYAG | 400 | 92 | (7) |
|  |  |  | R: TGTAAGTTCGCCCTTTGTCCAT | 400 |  |  |
|  |  |  | P: FAM-CAATGACAGACGCCTTCCATGGTAATAGGG-BHQ1 | 250 |  |  |
| 4 | *A. phagocytophilum*^b^ | 23S rRNA | F: TTTTCTGCAATACTTGTCGTGAAAT | 400 | 73 | (3) |
|  |  |  | R: TCGGAATGTTACCGGGTGTT | 400 |  |  |
|  |  |  | P: VIC-CACTCCYGCCATTACCACCAGCCAAA-BHQ1 | 200 |  |  |
|  | CCHFV^a^ | NP | F: CAAAGAAACACGTGCCGCTT | 500 | 76 | (2) |
|  |  |  | R: ATTCACCTCGATTTTGTTTTCCAT | 500 |  |  |
|  |  |  | P: Cy5-ACGCCCACAGTGTTCTCTTGAGTGTTAGCA-BHQ2 | 250 |  |  |
|  | *Rickettsia* spp.^b^ | 23S rRNA | F: AGCTTGCTTTTGGATCATTTGG | 400 | 110 | (2) |
|  |  |  | R: TTCCTTGCCTTTTCATACATCTAGT | 400 |  |  |
|  |  |  | P: FAM-CCTGCTTCTATTTGTCTTGC-MGB | 250 |  |  |
| 5 | *C. burnetii*^b^ | *IS1111* | F: CCGATCATTTGGGCGCT | 900 | 63 | (2) |
|  |  |  | R: CGGCGGTGTTTAGGC | 900 |  |  |
|  |  |  | P: VIC-TTAACACGCCAAGAAACGTATCGCTGTG -BHQ1 | 250 |  |  |
|  | *Ehrlichia* spp.^b^ | 16S rRNA | F: TACAAAGATAAAATCCTCACATTT | 900 | 156 | (3) |
|  |  |  | R: ATTCGTAGATATTAGGAGGAACAC | 900 |  |  |
|  |  |  | P: Cy5-CCTGTTTGCTCCCCACGCTT-BHQ2 | 250 |  |  |
|  | *T. gondii*^c^ | 18S rRNA | F: GGTGGTCCTCAGGTGAT | 900 | 116 | (8) |
|  |  |  | R: CCACGGTAGTCCAATACAGTA | 900 |  |  |
|  |  |  | P: FAM-ATCGCGTTGACTTCGGTCTGCGAC-BHQ1 | 250 |  |  |
| 6 | Bacteriophage MS2^d^ | g1 | F: TGGCACTACCCCTCTCCGTATTCAC | 600 | 99 | (2) |
|  |  |  | R: GTACGGGCGACCCCACGATGAC | 600 |  |  |
|  |  |  | P: FAM-CACATCGATAGATCAAGGTGCCTACAAGC-BHQ1 | 250 |  |  |
|  | SGLV^a^ | S | F: GGAGAAGGACGCTGAGATTG | 600 | 157 | This study |
|  |  |  | R: CTTGGACCACTTCATGTTCTTG | 600 |  |  |
|  |  |  | P: Cy5-CTCATGCACGAACGGCTGAAG-BHQ2 | 300 |  |  |
|  | *Hepatozoon* spp.^c^  / *Theileria* spp.^c^ | 18S rRNA | F: TGAGAAAATTAGAGTGCTYAAAGC | 600 | 164 | (9)  Modified |
|  |  |  | R: CTAAGAATTTCACCTCTGACAGTTA | 600 |  |  |
|  |  |  | P: VIC- CCRAAGTAATGGTTAATAGGAACAGTT-MGB | 300 |  |  |
| 7 | NSDV^a^ | L | F: ATGTGGACGGCAAAATAACAAC | 600 | 147 | This study |
|  |  |  | R: CCTTATCCTCTGAGATCCACCA | 600 |  |  |
|  |  |  | P: Cy5-CACATCAACACCTGCGGACA-BHQ2 | 200 |  |  |
|  | TAMV^a^ | S | F: GGGACGAATGGGCAAAG | 500 | 74 | This study |
|  |  |  | R: CCTCTGTATCATGCCTGCAC | 500 |  |  |
|  |  |  | P: VIC-TCAACAAGTCAGGACACAGCCT-MGB | 200 |  |  |
|  | WELV^a^ | S | F: AACCGATGGGAAAGTGACTG | 500 | 264 | (10) |
|  |  |  | R: GAACTTGAGGGCGTTGACAT | 500 |  |  |
|  |  |  | P: FAM-ACCCTGTGCTCTGAAAGCAT-MGB | 200 |  |  |
| 8 | *Babesia* spp.^b^ | 18S rRNA | F: TGACAAGAAATAACAATACAGG | 700 | 157 | This study |
|  |  |  | R: AGCTTTTTAACTGCAACAAG | 700 |  |  |
|  |  |  | P: FAM-CTCACCAGAGTAMCAATT-MGB | 200 |  |  |
|  | *B. duncani*^c^ | 18S rRNA | F: TGGTGGAGTGATTTGTCTG | 600 | 108 | This study |
|  |  |  | R: CAAAGTCCCTCTAAGAAGCAA | 600 |  |  |
|  |  |  | P: FAM-TGCTAAATAGCAGCTGAGAATA-MGB | 200 |  |  |
|  | *B. microti*^c^ | ITS | F: AGGTTTTCTGTGTGTGCATT | 700 | 94 | This study |
|  |  |  | R: ACTATCTACCACACAAACAAGT | 700 |  |  |
|  |  |  | P: ROX-TCACGCAGAAGGTTCTTTGA-MGB | 250 |  |  |
|  | NOMV^a^ | L | F: GCTCTGAAGAAGTACATTGA | 700 | 109 | (11) |
|  |  |  | R: GCATGTGTGAYGTTTCAG | 700 |  |  |
|  |  |  | P: VIC-CTGACTGGAGACGCACACGAC-BHQ2 | 200 |  |  |
|  | TcTV-1^a^ | N | F: ACTAGGGCAGAGAATGATG | 700 | 117 | This study |
|  |  |  | R: GAGTCCTCGTTCAACCAT | 700 |  |  |
|  |  |  | P: Cy5-CCAATAAAGGAGTTTGCATGGA-MGB | 200 |  |  |

**Note**: ^a^Viruses; ^b^Bacteria; ^c^Protozoa; ^d^Internal control.

**Abbreviations**: F, forward primer; R, reverse primer; P, TaqMan probe; *A*. *phagocytophilum*, *Anaplasma phagocytophilum*; ALSV, Alongshan virus; Bacteriophage MS2, Emesvirus zinderi; BJNV, Beiji-nairovirus; *C*. *burnetii*, *Coxiella burnetii*; CCHFV, Crimean-Congo hemorrhagic fever virus; *F*. *tularensis*, *Francisella tularensis*; JMTV, Jingmen tick virus; NOMV, Nuomin virus; NSDV, Nairobi sheep disease virus; SFTSV, Severe fever with thrombocytopenia syndrome virus; SGLV, Songling virus; *T*. *gondii*, *Toxoplasma* *gondii*; TBEV, Tick-borne encephalitis virus; TcTV-1, Tacheng tick virus-1; TAMV, Tamdy virus; WELV, Wetland virus; XCV, Xue-Cheng virus; YEZV, Yezo virus.

**Reference**

1.	Wang T, Guo Y, Xu Y, Sun H, Peng P, Qin S, et al. Geographical distribution and characterization of Jingmen tick virus in wild boars in China. Virol Sin. 2025;40(1):137-40. https://doi.org/10.1016/j.virs.2024.12.011

2.	Liu J, Ochieng C, Wiersma S, Ströher U, Towner JS, Whitmer S, et al. Development of a TaqMan Array Card for Acute-Febrile-Illness Outbreak Investigation and Surveillance of Emerging Pathogens, Including Ebola Virus. J Clin Microbiol. 2016;54(1):49-58. https://doi.org/10.1128/jcm.02257-15

3.	Peñataro Yori P, Paredes Olórtegui M, Schiaffino F, Colston JM, Pinedo Vasquez T, Garcia Bardales PF, et al. Etiology of acute febrile illness in the peruvian amazon as determined by modular formatted quantitative PCR: a protocol for RIVERA, a health facility-based case-control study. BMC Public Health. 2023;23(1):674. https://doi.org/10.1186/s12889-023-15619-6

4.	Yu M, Fu X, Zhu Y, Li M, Wu M, Zhou E, et al. TaqMan qPCR and IgM Detection in Samples of Patients with Tick-Borne Encephalitis Virus Infection in Northeast China. Int J Gen Med. 2024;17:3745-53. https://doi.org/10.2147/ijgm.S475450

5.	Ariizumi T, Tabata K, Itakura Y, Kobayashi H, Hall WW, Sasaki M, et al. Establishment of a lethal mouse model of emerging tick-borne orthonairovirus infections. PLoS Pathog. 2024;20(3):e1012101. https://doi.org/10.1371/journal.ppat.1012101

6.	Liu YT, Z. L. Li, X. F. Fu, S. H. Zhou, J. He, Y. Wang, H. Y. Establishment of multiplex real-time quantitative RT-PCR for rapid detection of Alongshan virus and Songling virus. Chinese Journal of Microbiology and Immunology. 2021;41(4):316-22. https://doi.org/10.3760/cma.j.cn112309-20200826-00448

7.	Sun Y, Liang M, Qu J, Jin C, Zhang Q, Li J, et al. Early diagnosis of novel SFTS bunyavirus infection by quantitative real-time RT-PCR assay. J Clin Virol. 2012;53(1):48-53. https://doi.org/10.1016/j.jcv.2011.09.031

8.	Moore CC, Jacob ST, Banura P, Zhang J, Stroup S, Boulware DR, et al. Etiology of Sepsis in Uganda Using a Quantitative Polymerase Chain Reaction-based TaqMan Array Card. Clin Infect Dis. 2019;68(2):266-72. https://doi.org/10.1093/cid/ciy472

9.	Yang Y, Mao Y, Kelly P, Yang Z, Luan L, Zhang J, et al. A pan-Theileria FRET-qPCR survey for Theileria spp. in ruminants from nine provinces of China. Parasit Vectors. 2014;7:413. https://doi.org/10.1186/1756-3305-7-413

10.	Zhang XA, Ma YD, Zhang YF, Hu ZY, Zhang JT, Han S, et al. A New Orthonairovirus Associated with Human Febrile Illness. N Engl J Med. 2024;391(9):821-31. https://doi.org/10.1056/NEJMoa2313722

11.	Hu K, Liu T, Xu W, Liu Z, Wang Z, Ma J, et al. Real-time quantitative reverse transcription PCR assay for the detection of Nuomin virus - An emerging tick-borne virus. J Virol Methods. 2024;330:115032. https://doi.org/10.1016/j.jviromet.2024.115032

Supplementary Table S5. Nucleic acids from intended target pathogens used to evaluate the inclusivity of the TBP Panel

| Category | Pathogen | Strains (n) |
| --- | --- | --- |
| Bacteria | *Anaplasma phagocytophilum* | 2 |
|  | *Bartonella grahamii* | 5 |
|  | *Bartonella* spp. | 5 |
|  | *Borrelia* spp. | 10 |
|  | *Coxiella burnetii* | 3 |
|  | *Ehrlichia* spp. | 10 |
|  | *Francisella tularensis subsp novicida* | 1 |
|  | *Francisella tularensis subsp tularensis* | 1 |
|  | *Spotted fever group rickettsiae* | 2 |
|  | *Typhus group rickettsiae* | 3 |
|  | *Rickettsia* spp. | 5 |
| Parasite | *Hepatozoon* *canis* | 3 |
|  | *Hepatozoon* spp. | 7 |
|  | *Theileria cervi* | 3 |
|  | *Theileria luwenshuni* | 5 |
|  | *Theileria orientalis* | 3 |
|  | *Babeisia bigemina* | 3 |
|  | *Babesia micioti* | 3 |
|  | *Babesia occultans* | 1 |
|  | *Babesia ovata* | 2 |
|  | *Babesia pecorum* | 1 |
|  | *Babesia venatorum* | 1 |
|  | *Babesia vogeli* | 2 |
| Virus | Alongshan virus | 2 |
|  | Beiji nairovirus | 2 |
|  | Jingmen tick virus | 5 |
|  | Severe fever with thrombocytopenia syndrome virus | 2 |
|  | Nairobi sheep disease virus | 3 |
|  | Nuomin virus | 1 |
|  | Yezo virus | 1 |

Supplementary Table S6. Nucleic acids from non-target pathogens used to evaluate the specificity of the TBP Panel

| Category | Pathogen | Strains (n) |
| --- | --- | --- |
| Bacteria | *Acinetobacter baumannii* | 2 |
|  | *Aeromonas hydrophila* | 1 |
|  | *Brucella melitensis* | 2 |
|  | *Coagulase-negative staphylococci* | 1 |
|  | *Enterobacter aerogenes* | 1 |
|  | *Enterobacter cioacae* | 4 |
|  | *Enterobacter cioacae* complex | 2 |
|  | *Enterococcus faecalis* | 1 |
|  | *Escherichia coli* | 6 |
|  | *Klebsiella pneumoniae* | 1 |
|  | Methicillin-resistant *staphylococcus aureus* | 3 |
|  | *Moraxella catarrhalis* | 1 |
|  | *Nocardia asteroides* | 2 |
|  | *Orientia tsutsugamushi* | 1 |
|  | *Proteus mirabilis* | 1 |
|  | *Proteus penneri* | 1 |
|  | *Proteus vulgaris* | 1 |
|  | *Providencia rettgeri* | 1 |
|  | *Pseudomonas aeruginosa* | 2 |
|  | *Pseudomonas stutzeri* | 1 |
|  | *Rothia dentocariosa* | 1 |
|  | *Serratia marcescens* | 1 |
|  | *Shigella flexneri* | 1 |
|  | *Staphylococcus aureus* | 10 |
|  | *Staphylococcus epidermidis* | 3 |
|  | *Staphylococcus haemolyticus* | 3 |
|  | *Staphylococcus hominis* | 1 |
|  | *Staphylococcus simulans* | 1 |
|  | *Stenotrophomonas maltophilia* | 1 |
|  | *Streptococcus agalactiae* | 1 |
|  | *Streptococcus pneumoniae* | 1 |
| Fungi | *Aspergillus fumigatus* | 1 |
|  | *Candida albicans* | 1 |
|  | Adenovirus | 2 |
|  | Cytomegalovirus | 1 |
|  | Hepatitis B virus | 4 |
|  | Hemorrhagic fever with renal syndrome virus | 2 |
|  | Human herpesvirus 5 | 5 |
|  | Human herpesvirus 6 | 5 |
|  | Human herpesvirus 7 | 5 |
|  | Human metapneumovirus | 1 |
|  | Japanese encephalitis virus | 1 |
|  | Rhinovirus | 1 |
|  | Influenza A virus | 2 |
|  | Measles | 2 |
|  | Norovirus | 1 |
|  | Severe acute respiratory syndrome coronavirus 2 | 1 |

Supplementary Table S7. MIQE 2.0 checklist for this study

|  | **PROVIDED** | **DESCRIPTION / JUSTIFICATION** |
| --- | --- | --- |
| **1. REAGENT PREPARATION** | | |
| Bioinformatics tools and versions and settings used to design assays | Yes | Primer design was performed using Primer3 (v. 4.1.0). In silico validation for secondary structures and specificity was conducted using the IDT OligoAnalyzer™ tool and BLASTn (NCBI) |
| Official gene symbol, species and sequence accession number | Yes | Provided in Table S4 |
| Location of amplicon | Yes | Provided in Table S4 |
| Amplicon length | Yes | Provided in Table S4 |
| Primer and probe sequences | Yes | Provided in Table S4 |
| Location and identity of any modifications | Yes | Provided in Table S4 |
| Manufacturer of oligonucleotides | Yes | All oligonucleotides were synthesized by Sangon Biotech (Wuhan, China) |
| Details of optimization performed | Yes | Primer concentrations and annealing temperatures were optimized for each multiplex assay to ensure high amplification efficiency and prevent non-specific amplification |
| **2. SAMPLE PREPARATION** | | |
| Detailed description of sample types and numbers | Yes | The study included 2,333 ticks (in 225 pools), 59 rodent liver samples, and 553 febrile patient whole blood samples. Details are in the 'MATERIALS AND METHODS' section |
| Sampling procedure (including time to storage) | Yes | Ticks were collected by flagging vegetation and removal from domestic animals. Human samples were collected by venipuncture. Rodent samples were collected post-euthanasia. See 'Materials and Methods' for details |
| Sample aliquoting, storage conditions and duration | Yes | All samples were stored at -80°C until nucleic acid extraction |
| Description of extraction method including amount of sample processed | Yes | Two distinct extraction protocols were used based on the sample cohort and matrix  1. Automated Extraction: For the acute febrile illness patient samples, total nucleic acids were extracted from 2 mL of whole blood using the MAGABio Virus DNA / RNA Maxi Purification Kit Ⅱ on a Fully Automatic Nucleic Acid Extraction and Purification System (Bioer, Hangzhou, China)  2. Manual Extraction: Manual nucleic acid extraction was performed using the Biospin Virus DNA / RNA Extraction Kit (Bioer, Hangzhou, China) for the following samples: Tick pools, with pool sizes standardized by number and life stage as follows: 1 - 10 engorged ticks, 10 - 20 free-living adults / nymphs, or 50 - 80 free-living larvae; 200 μL of whole blood from fever of unknown origin patients; Half the volume of each rodent liver aliquot |
| Source and amount of spike-in nucleic acids added | Yes | 10^6^ of MS2 bacteriophage was added to each sample prior to extraction as an internal process control |
| Volume of elution buffer used to elute/resuspend nucleic acids | Yes | The elution volume was specific to the extraction method used. For the magnetic bead-based extraction samples, nucleic acids were eluted in 300 µL elution buffer. For the column-based extraction samples, nucleic acids were eluted in 100 µL elution buffer |
| Number of extraction replicates | Yes | A single nucleic acid extraction was performed for each individual sample or pool |
| Extraction blanks and percent yield included | Yes | An extraction blank (nuclease-free water) was included with each batch of extractions to monitor for cross-contamination. The percent yield of nucleic acids was not determined |
| Method to evaluate quality and quantity of nucleic acids | Yes | The concentration and purity (A260 / A280 ratio) of the eluted nucleic acids were assessed using a NanoDrop spectrophotometer (Thermo Fisher Scientific) |
| Storage conditions: temperature, concentration, duration, buffer, aliquots | Yes | Eluted nucleic acids were stored at -80°C in single-use aliquots. The template was used for qPCR undiluted. The precise storage duration for each sample is not available as they were collected over time |
| Clear description of dilution steps used to prepare working template solution | Yes | The nucleic acid eluate was used directly as the working template solution without any further dilution steps |
| Template modification (digestion, sonication, pre-amplification, DNAse treatment etc.) | No | NA. No template modification steps were performed after extraction |
| Purification after modification | Yes | NA, as no template modifications were performed |
| **3. REVERSE TRANSCRIPTION** | | |
| cDNA priming method and primer concentration | Yes | Reverse transcription was performed using random hexamers and/or gene-specific primers provided with the HiScript III U+ One Step qRT-PCR Probe Master Mix kit |
| One or two-step protocol (include reaction details for two-step) | Yes | A one-step RT-qPCR protocol was used, where reverse transcription and qPCR amplification occur in the same tube |
| Amount of RNA used per reaction | Yes | 2 µL of the total nucleic acid eluate was used as template for each reaction. The specific concentration of RNA was not normalized prior to the reaction |
| Detailed reaction components and conditions | Yes | The reverse transcription step was performed at 55°C for 15 minutes, followed by enzyme inactivation, as per the manufacturer's protocol for the HiScript III U+ One Step qRT-PCR Probe Master Mix kit |
| Estimated copies measured with and without addition of RT | No | NA. This control is typically for gene expression studies. For this multiple panel targeting both DNA and RNA pathogens, gDNA contamination is not a confounding factor for the primary analysis |
| Manufacturer of reagents, catalog number and lot number | Yes | HiScript III U+ One Step qRT-PCR Probe Master Mix (Vazyme, Nanjing, China; Cat. No.: q611-03; Lot No.: 7E0190D5) |
| Storage of cDNA: temperature, concentration/dilution, duration, buffer, aliquots | No | NA. A one-step protocol was used, so no cDNA was stored |
| **4. qPCR PROTOCOL** | | |
| Template treatment (initial heating or chemical denaturation) | Yes | Initial template denaturation and enzyme activation were performed at 95°C for 30 seconds |
| Primer and probe concentration in the reaction and source | Yes | All oligonucleotides were synthesized by Sangon Biotech (Wuhan, China). The final concentrations used in each reaction are detailed in Table S4 |
| Polymerase identity and concentration, Mg2+ and dNTP concentrations | Yes | These components are proprietary to the HiScript III U+ One Step qRT-PCR Probe Master Mix and were used as supplied according to the manufacturer's recommendations |
| Buffer / kit (manufacturer, catalog number and lot number) | Yes | HiScript III U+ One Step qRT-PCR Probe Master Mix (Vazyme, Nanjing, China; Cat. No.: q611-03; Lot No.: 7E0190D5) |
| Complete thermocycling parameters including reaction volume | Yes | Reactions were performed in a 10 µL volume. The thermal cycling parameters consisted of an initial reverse transcription at 55°C for 15 minutes, followed by polymerase activation at 95°C for 30 seconds, and then 45 cycles of denaturation at 95°C for 10 seconds and annealing/extension at 60°C for 1 minute |
| Manufacturer and type of qPCR instrument | Yes | All reactions were run on an Applied Biosystems 7500 Real-Time PCR System (Thermo Fisher Scientific) |
| **5. DATA ANALYSIS** | | |
| Storage and submission of raw fluorescence data using RDES or RDML | No | NA. Raw fluorescence and Cq data are not publicly archived but are available from the corresponding author upon reasonable request |
| Identity of standards (synthetic, plasmid, genomic, IVT, mRNA etc.) and method of quantification | Yes | A synthetic plasmid containing target sequences for all Target was used as the standard. The plasmid was quantified by the manufacturer based on its known molecular weight and concentration, and copy number was calculated accordingly |
| Method of baseline correction and Cq determination | Yes | Baseline correction and Cq determination were initially performed using the automated settings of the analysis software. All amplification curves were then manually inspected, and the baseline or threshold was adjusted where necessary to ensure accurate Cq values |
| qPCR analysis program (source, version) | Yes | Data were analyzed using Design and Analysis Software v2.6 (Thermo Fisher Scientific) |
| Details of positive and negative controls | Yes | Each qPCR run included a positive control (plasmid standard at 10^6^ copies / mL) and a no-template control (NTC) using nuclease-free water |
| Frequency and Cq of negative controls | Yes | NTCs were included in every run and were consistently negative (Cq ≥ 40) |
| Examples of positive and negative results | Yes | Positive samples were characterized by a clear sigmoidal amplification curve with a Cq value below the established cutoff. Negative samples, including the no-template controls, showed no detectable amplification signal within 40 cycles. |
| PCR efficiency estimation and method for its determination | Yes | Amplification efficiency (E) was calculated from the slope of the 10-fold serial dilution standard curve using the formula: E = (10^-1^ / slope − 1) × 100. The 95% CI of the efficiency was also calculated |
| Method of target quantity calculation | Yes | This study focused on qualitative detection (presence / absence). Samples were classified as positive or negative based on the established optimal Cq cutoff for each target |
| Description of replicates | Yes | All samples, standards, and controls were run in technical duplicate |
| Repeatability (intra-experiment variation) | Yes | Repeatability was assessed as intra-assay variation. The coefficient of variation (CV) was calculated from 20 replicate tests of standard plasmids (at 10^5^ and 10^6^ copies/mL in a whole blood matrix) conducted within a single experimental run |
| Reproducibility (inter-experiment/user/lab etc. variation) | Yes | Reproducibility was assessed by evaluating inter-experiment and inter-operator variation within a single laboratory. The CV was calculated from a total of 20 replicates for each standard plasmid concentration. The experiment consisted of three independent runs conducted over a five-day period, performed by two different operators |
| Limit of detection calculated? | Yes | The 99% limit of detection (LOD_99%_) and its 99% CI were calculated for each target in both blood and tick matrices using probit regression analysis |
| Dynamic range (limits of quantification) | Yes | The linear dynamic range of the assays was established over a 10-fold serial dilution of the plasmid standard, spanning from 10^8^ to 10^4^ copies / mL, with high linearity (R^2^ > 0.99) observed across this range |
| Method of validation of reference genes | No | NA. This study involves pathogen detection, not gene expression analysis, so no reference genes were used |
| Description of normalization method / calculation of normalized expression | No | NA. This study reports qualitative detection results and does not involve normalization of expression levels. |
| Statistical methods used for analysis | Yes | Statistical methods included descriptive statistics, linear regression (for standard curves), probit regression (for LOD), and receiver operating characteristic (ROC) curve analysis. See the 'Statistical Analysis' section for full details |
| Choice of significance level and calculation of statistical power | No | NA. A formal power analysis was not performed as this was a validation and descriptive surveillance study |

**Abbreviations**: NA, Not Applicable.

Supplementary Table S8. Primer sequences used for the nested / semi-nested PCR validation assays

| Assay | Pathogen | Target | Sequence (5′ - 3′) | Sizes (bp) | T_a_ (℃) | Reference |
| --- | --- | --- | --- | --- | --- | --- |
| 1 | BJNV | S | Outer-F: TGCTGGTGATGAGACCTCTG | 791 | 56 | (1) |
|  |  |  | Outer-R: CTGAGGTGCTTGGTGTGGA |  |  |  |
|  |  |  | Inner-F: TGACAGCCCTCAGTGGAGAA | 581 | 52 | This study |
|  |  |  | Inner-R: TGGGACAACCTCAACATTCA |  |  |  |
|  | JMTV | Segment 2 | Outer-F: GGAGAGAACGATGTCCAAATAAGGCAGA | 500 | 60 | (2) |
|  |  |  | Outer-R: GTGTATGTTAGGACGATGACAAAGACCCG |  |  |  |
|  |  |  | Inner-F: GGCTCACCATTAGAGTCACGGGAAG | 351 | 60 | (2) |
|  |  |  | Inner-R: TTGGTAAGTTGTTGACCACTAGATCCACC |  |  |  |
|  | XCV | S | Outer-F: CTAGGCTTCGACTTGCATGT | 599 | 54 | This study |
|  |  |  | Outer-R: TGCTGCTTGGTGTTCTTCTT |  |  |  |
|  |  |  | Inner-F: GCACATCCTCTCCTAGCATG | 429 | 54 | This study |
|  |  |  | Inner-R: TGAGCTTCATGTCTTCCAGC |  |  |  |
| 2 | *Bartonella* spp. | *ssrA* | Outer-F: GCTATGGTAATAAATGGACAATGAAATAA | 301 | 50 | (3) |
|  |  |  | Outer-R: GCTTCTGTTGCCAGGTG |  |  |  |
|  |  |  | Inner-F: GGCTAAATNAGTAGTTGCAAAYGACA | 133 | 54 | (4) |
|  |  |  | Inner-R: GCTTCTGTTGCCAGGTG |  |  |  |
|  | *Borrelia* spp. | 16S rRNA | Outer-F: CAACAGATTCCACCCTTAC | 699 | 48 | This study |
|  |  |  | Outer-R: ATAACGAAGAGTTTGATCCTG |  |  |  |
|  |  |  | Inner-F: CAGATTCCACCCTTACACCA | 488 | 52 | This study |
|  |  |  | Inner-R: AGCTTCGCTTRTAGATGAGTCT |  |  |  |
|  | TBEV | E | Outer-F: GTBACCATAACAGCYGAGG | 449 | 53 | (5) |
|  |  |  | Outer-R: CCRTAGTHTCCCATGGTCAAG |  |  |  |
|  |  |  | Inner-F: GCCTDCACGCMAAGCTRTCG | 361 | 54 | (5) |
|  |  |  | Inner-R: CCRTAGTHTCCCATGGTCAAG |  |  |  |
|  | YEZV | L | Outer-F: ACCCCACATACTCTCTAYAACGA | 286 | 50 | (6) |
|  |  |  | Outer-R: CTGAAGCCTCCCTAAGTGC |  |  |  |
|  |  |  | Inner-F: ACCCCACATACTCTCTAYAACGA | 255 | 45 | (6) |
|  |  |  | Inner-R: TACTGTGGCCTTGATCTCACT |  |  |  |
| 3 | ALSV | NS5 | Outer-F: CCCTCGAAAGGYTATGACA | 428 | 52 | (7) |
|  |  |  | Outer-R: CTTCCCAGTCTTGACCTGC |  |  |  |
|  |  |  | Inner-F: CCCTCGAAAGGYTATGACA | 333 | 52 | (7) |
|  |  |  | Inner-R: GTTGCATCATCCAYGCATC |  |  |  |
|  | *F. tularensis* | *tul4* | Outer-F: GCTGTATCATCATTTAATAAACTGCTG | 410 | 51 | (8) |
|  |  |  | Outer-R: TTGGGAAGCTTGTATCATGGCACT |  |  |  |
|  |  |  | Inner-F: GCTGTATCATCATTTAATAAACTGCT | 344 | 50 | This study |
|  |  |  | Inner-R: CTTGTGGGTTATTATTGTATGYTG |  |  |  |
|  | SFTSV | L | Outer-F: TCCAATGAYGCCAAGAAGT | 867 | 56 | This study |
|  |  |  | Outer-R: CWGGATCTGGGTCYGATAT |  |  |  |
|  |  |  | Inner-F: CCAGCYAAATTCCACAGATT | 698 | 56 | This study |
|  |  |  | Inner-R: TGTATRAGGTAGGTAAGAGAGAAT |  |  |  |
| 4 | *A. phagocytophilum* | 16S rRNA | Outer-F: TTGAGAGTTTGATCCTGGCTCAGAACG | 653 | 55 | (9) |
|  |  |  | Outer-R: CACCTCTACACTAGGAATTCCGCTATC |  |  |  |
|  |  |  | Inner-F: GTCGAACGGATTATTCTTTATAGCTTG | 389 | 55 | (9) |
|  |  |  | Inner-R: TATAGGTACCGTCATTATCTTCCCTAC |  |  |  |
|  | CCHFV | S | Outer-F: TGGACACYTTCACAAACTC | 536 | 54 | (10) |
|  |  |  | Outer-R: GRYRAAYTCCCTRCACCA |  |  |  |
|  |  |  | Inner-F: GAATGTGCATGGGTTAGCTC | 260 | 54 | (10) |
|  |  |  | Inner-R: GACATCACAATTTCACCAGG |  |  |  |
|  | *Rickettsia* spp. | 23S-5S rRNA ITS | Outer-F: GATAGGTCRGRTGTGGAAGCAC | 388 - 467 | 56 | (11) |
|  |  |  | Outer-R: TCGGGAYGGGATCGTGTGTTTC |  |  |  |
|  |  |  | Inner-F: TGTGGAAGCACAGTAATGTGTG | 350 | 54 | (11) |
|  |  |  | Inner-R: TCGTGTGTTTCACTCATGCT |  |  |  |
| 5 | *C. burnetii* | *IS1111* | Outer-F: TATGTATCCACCGTAGCCAGTC | 687 | 54 | (12) |
|  |  |  | Outer-R: CCCAACAACAACCTCCTTATTC |  |  |  |
|  |  |  | Inner-F: AAGCGTGTGGAGGAGCGAACC | 440 | 54 | (13) |
|  |  |  | Inner-R: CTCGTAATCACCAATCGCTTCGTC |  |  |  |
|  | *Ehrlichia* spp. | 16S rRNA | Outer-F: TCGGAATTATTGGGCGTAA | 453 | 54 | This study |
|  |  |  | Outer-R: AGGGATACGACCTTCATGTCA |  |  |  |
|  |  |  | Inner-F: TACAAAGATAAAATCCTCACATTT | 156 | 48 | (4) |
|  |  |  | Inner-R: ATTCGTAGATATTAGGAGGAACAC |  |  |  |
|  | *T. gondii* | 18S rRNA | Outer-F: CCATGCATGTCTAAGTATAAGC | 314 | 55 | (14) |
|  |  |  | Outer-R: GTTACCCGTCACTGCCAC |  |  |  |
|  |  |  | Inner-F: CTAAGTATAAGCTTTTATACGGC | 290 | 54 | (14) |
|  |  |  | Inner-R: TGCCACGGTAGTCCAATAC |  |  |  |
| 6 | SGLV | S | Outer-F: ACATGGCACCTGTGTATGAGTG | 856 | 53 | (15) |
|  |  |  | Outer-R: ACAAACGGGGATTCCTTCA |  |  |  |
|  |  |  | Inner-F: CAAGGAGTACGAAAGCCTGAA | 674 | 53 | (15) |
|  |  |  | Inner-R: AGGACAGTGTTGGGAAGAGC |  |  |  |
|  | *Hepatozoon* spp.  */ Theileria* spp. | 18S rRNA | Outer-F: GAAACGGCTACCACATCT | 778 | 55 | (16) |
|  |  |  | Outer-R: AGTTTCCCCGTGTTGAGT |  |  |  |
|  |  |  | Inner-F: TTAAACCTCTTCCAGAGT | 509 | 55 | (16) |
|  |  |  | Inner-R: AAACTGACGACCTCCAAT |  |  |  |
| 7 | NSDV | L | Outer-F: GTTGTTGCTGTYCTACTGTC | 849 | 54 | This study |
|  |  |  | Outer-R: CTTCCATGCTGTACTGTGA |  |  |  |
|  |  |  | Inner-F: TCAGTYATGAGTCCAATAAGT | 450 | 54 | This study |
|  |  |  | Inner-R: CTTTATCTCAATCAGAAGCTG |  |  |  |
|  | TAMV | S | Outer-F: GCGTTATTCTTGGGCTATT | 671 | 49 | (17) |
|  |  |  | Outer-R: ATTTCGCCATTGACTTTGT |  |  |  |
|  |  |  | Inner-F: TTAAGACCTGGTGCGATGA | 328 | 52 | This study |
|  |  |  | Inner-R: GCCCATATTTGATGTCCTCC |  |  |  |
|  | WELV | S | Outer-F: CTGGGAAACCGATGGGAAAG | 682 | 54 | (18) |
|  |  |  | Outer-R: CAAGGTCTGGGACGCAAACTA |  |  |  |
|  |  |  | Inner-F: CTGGGAAACCGATGGGAAAG | 454 | 56 | (18) |
|  |  |  | Inner-R: CATCCTGAATGGAGGGTCGG |  |  |  |
| 8 | *Babesia.* spp. | 18S rRNA | Outer-F: AATTACCCAATCCTGACACAGG | 506 | 53 | (19) |
|  |  |  | Outer-R: TTTCGCAGTAGTTCGTCTTTAACA |  |  |  |
|  |  |  | Inner-F: GACACAGGGAGGTAGTGACAAGA | 400 | 55 | (19) |
|  |  |  | Inner-R: CCCAACTGCTCCTATTAACCATTAC |  |  |  |
|  | NoMV | N | Outer-F: TAGATCGATACCRCACTCYAG | 407 | 49 | (20) |
|  |  |  | Outer-R: CTTGWATTATACTYCGTGCWC |  |  |  |
|  |  |  | Inner-F: TAGATCGATACCRCACTCYAG | 358 | 50 | (20) |
|  |  |  | Inner-R: YCTTGCCTCAATTTTATGCTC |  |  |  |
|  | TcTV-1 | S | Outer-F: CGGCTCAGTGTTTATCCC | 423 | 55 | (21) |
|  |  |  | Outer-R: GTTGACCTCAGCGGTTGT |  |  |  |
|  |  |  | Inner-F: CTTCTCACTTTCTCGGATGCTT | 328 | 50 | (21) |
|  |  |  | Inner-R: TTCCTTTTAGTGCTTCGTAGTT |  |  |  |

**Abbreviations**: T_a_, Annealing temperature; F, forward primer; R, reverse primer; Outer, outer primers used for the first PCR round; Inner, nested primers used for the second PCR round.

**Reference**

1.	Wang YC, Wei Z, Lv X, Han S, Wang Z, Fan C, et al. A new nairo-like virus associated with human febrile illness in China. Emerg Microbes Infect. 2021;10(1):1200-8. https://doi.org/10.1080/22221751.2021.1936197

2.	Yu ZM, Chen JT, Qin J, Guo JJ, Li K, Xu QY, et al. Identification and characterization of Jingmen tick virus in rodents from Xinjiang, China. Infect Genet Evol. 2020;84:104411. https://doi.org/10.1016/j.meegid.2020.104411

3.	Diaz MH, Bai Y, Malania L, Winchell JM, Kosoy MY. Development of a novel genus-specific real-time PCR assay for detection and differentiation of Bartonella species and genotypes. J Clin Microbiol. 2012;50(5):1645-9. https://doi.org/10.1128/jcm.06621-11

4.	Peñataro Yori P, Paredes Olórtegui M, Schiaffino F, Colston JM, Pinedo Vasquez T, Garcia Bardales PF, et al. Etiology of acute febrile illness in the peruvian amazon as determined by modular formatted quantitative PCR: a protocol for RIVERA, a health facility-based case-control study. BMC Public Health. 2023;23(1):674. https://doi.org/10.1186/s12889-023-15619-6

5.	Lu X, Wang D, Wang Z, Zhang L, Ji H, Wei F, et al. Establishment of a SYBR Green II real-time quantitative PCR assay for the detection of tick-borne encephalitis virus (TBEV) [in Chinese]. Chinese Journal of Veterinary Science. 2020;40(3):552-6. https://doi.org/10.16303/j.cnki.1005-4545.2020.03.18

6.	Lv X, Liu Z, Li L, Xu W, Yuan Y, Liang X, et al. Yezo Virus Infection in Tick-Bitten Patient and Ticks, Northeastern China. Emerg Infect Dis. 2023;29(4):797-800. https://doi.org/10.3201/eid2904.220885

7.	Xu W, Wang W, Li L, Li N, Liu Z, Che L, et al. Alongshan Virus Infection in Rangifer tarandus Reindeer, Northeastern China. Emerg Infect Dis. 2024;30(7):1434-7. https://doi.org/10.3201/eid3007.231219

8.	Wang Y, Qiao F, Cao J, Peng Y, Xia L. Discovery and tracking source of the new subgroup of Chinese Francisella tularensis type B [in Chinese]. Chinese Journal of Preventive Medicine. 2015;49(11):983-7.

9.	Yaxue Z, Hongtao J, Qiuyue W, Zhixin F, Hongwei G, Pengpeng L, et al. Molecular detection of Anaplasma phagocytophilum in Ixodid ticks in Hebei Province, China. Vector Borne Zoonotic Dis. 2011;11(10):1323-7. https://doi.org/10.1089/vbz.2010.0253

10.	Kong Y, Yan C, Liu D, Jiang L, Zhang G, He B, et al. Phylogenetic analysis of Crimean-Congo hemorrhagic fever virus in inner Mongolia, China. Ticks Tick Borne Dis. 2022;13(1):101856. https://doi.org/10.1016/j.ttbdis.2021.101856

11.	Kakumanu ML, Ponnusamy L, Sutton HT, Meshnick SR, Nicholson WL, Apperson CS. Development and Validation of an Improved PCR Method Using the 23S-5S Intergenic Spacer for Detection of Rickettsiae in Dermacentor variabilis Ticks and Tissue Samples from Humans and Laboratory Animals. J Clin Microbiol. 2016;54(4):972-9. https://doi.org/10.1128/jcm.02605-15

12.	Hoover TA, Vodkin MH, Williams JC. A Coxiella burnetti repeated DNA element resembling a bacterial insertion sequence. J Bacteriol. 1992;174(17):5540-8. https://doi.org/10.1128/jb.174.17.5540-5548.1992

13.	Choi KS, Hwang S, Kim MC, Cho HC, Park YJ, Ji MJ, et al. Molecular surveillance of zoonotic pathogens from wild rodents in the Republic of Korea. PLoS Negl Trop Dis. 2024;18(7):e0012306. https://doi.org/10.1371/journal.pntd.0012306

14.	Gao L, Rong X, He M, Zhang L, Li T, Wang W, et al. Metagenomic analysis of potential pathogens from blood donors in Guangzhou, China. Transfus Med. 2020;30(1):61-9. https://doi.org/10.1111/tme.12657

15.	Ma J, Lv XL, Zhang X, Han SZ, Wang ZD, Li L, et al. Author Correction: Identification of a new orthonairovirus associated with human febrile illness in China. Nat Med. 2021;27(5):926. https://doi.org/10.1038/s41591-021-01364-z

16.	Cao S, Zhang S, Jia L, Xue S, Yu L, Kamyingkird K, et al. Molecular detection of Theileria species in sheep from northern China. J Vet Med Sci. 2013;75(9):1227-30. https://doi.org/10.1292/jvms.13-0028

17.	Cui M, Zhou H, Zhang B, Carr MJ, Guo M, Shi W. Rapid detection of the emerging tick-borne Tamdy virus by TaqMan-based real-time reverse transcription PCR. J Virol Methods. 2022;305:114538. https://doi.org/10.1016/j.jviromet.2022.114538

18.	Yuan HX, Lv XL, Zhang MQ, Si GQ, Zhao ZJ, Han XH, et al. Genetic diversity and expanded epidemic area of novel tick-borne pathogen wetland virus in ticks, wild and domestic animals, and patient in China. Emerg Microbes Infect. 2025;14(1):2502003. https://doi.org/10.1080/22221751.2025.2502003

19.	Wei Q, Tsuji M, Zamoto A, Kohsaki M, Matsui T, Shiota T, et al. Human babesiosis in Japan: isolation of Babesia microti-like parasites from an asymptomatic transfusion donor and from a rodent from an area where babesiosis is endemic. J Clin Microbiol. 2001;39(6):2178-83. https://doi.org/10.1128/jcm.39.6.2178-2183.2001

20.	Hu K, Liu T, Xu W, Liu Z, Wang Z, Ma J, et al. Real-time quantitative reverse transcription PCR assay for the detection of Nuomin virus - An emerging tick-borne virus. J Virol Methods. 2024;330:115032. https://doi.org/10.1016/j.jviromet.2024.115032

21.	Liu X, Zhang X, Wang Z, Dong Z, Xie S, Jiang M, et al. A Tentative Tamdy Orthonairovirus Related to Febrile Illness in Northwestern China. Clin Infect Dis. 2020;70(10):2155-60. https://doi.org/10.1093/cid/ciz602

Supplementary Table S9. Comparison of Cq values between singleplex and multiplex qPCR formats for all assay targets^*^

| Assay | Pathogen | Optimized Component  (Fixed Parameter) | Tested Concentrations  (nM) | Corresponding Mean Cq Values | Optimal Conc.  (nM) | Final Multiplex Cq |
| --- | --- | --- | --- | --- | --- | --- |
| 1 | BJNV | Primer (Probe: 250 nM) | 400, 500, 600, 700, 800, 900, 1000 | 26.3, 26.7, 26.5, 26.1, 27.1, 27.4, 27.6 | 700 | 27.1 |
|  |  | Probe (Primer: 700 nM) | 150, 200, 250, 300, 350 | 26.7, 26.5, 26.6, 26.8, 27.1 | 200 |  |
|  | JMTV | Primer (Probe: 250 nM) | 400, 500, 600, 700, 800, 900, 1000 | 27.1, 27.3, 26.9, 26.8, 26.3, 26.5, 26.4 | 800 | 26.8 |
|  |  | Probe (Primer: 800 nM) | 150, 200, 250, 300, 350 | 26.2, 26.5, 26.0, 26.1, 26.9 | 250 |  |
|  | XCV | Primer (Probe: 250 nM) | 400, 500, 600, 700, 800, 900, 1000 | 27.3, 26.9, 26.8, 26.6, 26.5, 27.7, 27.9 | 800 | 26.9 |
|  |  | Probe (Primer: 800 nM) | 150, 200, 250, 300, 350 | 26.9, 27.5, 26.4, 26.6, 26.7 | 250 |  |
| 2 | *Bartonella* spp. | Primer (Probe: 250 nM) | 400, 500, 600, 700, 800, 900, 1000 | 25.9, 26.4, 26.5, 26.1, 26.7, 26.6, 26.4 | 400 | 26.7 |
|  |  | Probe (Primer: 400 nM) | 150, 200, 250, 300, 350 | 27.1, 26.9, 26.9, 27.1, 26.9 | 250 |  |
|  | *Borrelia* spp. | Primer (Probe: 250 nM) | 400, 500, 600, 700, 800, 900, 1000 | 26.4, 26.5, 26.9, 26.7, 26.5, 26.9, 27.0 | 400 | 26.6 |
|  |  | Probe (Primer: 400 nM) | 150, 200, 250, 300, 350 | 27.2, 27.1, 26.8, 26.9, 26.9 | 250 |  |
|  | TBEV | Primer (Probe: 250 nM) | 400, 500, 600, 700, 800, 900, 1000 | 26.5, 26.9, 26.5, 26.3, 26.4, 26.2, 26.5 | 400 | 26.8 |
|  |  | Probe (Primer: 400 nM) | 150, 200, 250, 300, 350 | 26.9, 26.6, 26.5, 26.1, 26.7 | 250 |  |
|  | YEZV | Primer (Probe: 250 nM) | 400, 500, 600, 700, 800, 900, 1000 | 27.0, 26.5, 27.1, 26.6, 26.3, 26.8, 27.4 | 500 | 27.1 |
|  |  | Probe (Primer: 500 nM) | 150, 200, 250, 300, 350 | 27.8, 26.9, 26.6, 26.5, 26.6 | 250 |  |
| 3 | ALSV | Primer (Probe: 250 nM) | 400, 500, 600, 700, 800, 900, 1000 | 25.8, 25.9, 25.9, 25.4, 25.9, 25.4, 25.8 | 400 | 25.8 |
|  |  | Probe (Primer: 400 nM) | 150, 200, 250, 300, 350 | 25.6, 25.4, 25.3, 25.4, 25.6 | 250 |  |
|  | *F*. *tularensis* | Primer (Probe: 250 nM) | 400, 500, 600, 700, 800, 900, 1000 | 26.8, 27.1, 27.4, 26.5, 26.6, 26.0, 26.1 | 400 | 26.9 |
|  |  | Probe (Primer: 400 nM) | 150, 200, 250, 300, 350 | 26.9, 26.8, 26.8, 26.9, 26.7 | 250 |  |
|  | SFTSV | Primer (Probe: 250 nM) | 400, 500, 600, 700, 800, 900, 1000 | 25.2, 25.6, 25.8, 25.2, 25.8, 25.5, 25.3 | 400 | 25.3 |
|  |  | Probe (Primer: 400 nM) | 150, 200, 250, 300, 350 | 25.3, 25.1, 25.0, 25.2, 25.1 | 250 |  |
| 4 | *A*. *phagocytophilum* | Primer (Probe: 250 nM) | 400, 500, 600, 700, 800, 900, 1000 | 26.1, 26.3, 26.2, 26.2, 26.1, 26.1, 26.0 | 400 | 26.8 |
|  |  | Probe (Primer: 400 nM) | 150, 200, 250, 300, 350 | 26.2, 26.1, 26.1, 26.8, 26.7 | 250 |  |
|  | CCHFV | Primer (Probe: 250 nM) | 400, 500, 600, 700, 800, 900, 1000 | 26.8, 26.4, 26.5, 26.7, 26.7, 26.7, 26.6 | 500 | 26.6 |
|  |  | Probe (Primer: 500 nM) | 150, 200, 250, 300, 350 | 27.1, 26.8, 26.5, 26.7, 26.8 | 250 |  |
|  | *Rickettsia* spp. | Primer (Probe: 250 nM) | 400, 500, 600, 700, 800, 900, 1000 | 26.1, 26.4, 26.5, 26.6, 26.9, 26.8, 27.4 | 400 | 26.9 |
|  |  | Probe (Primer: 400 nM) | 150, 200, 250, 300, 350 | 27.5, 27.2, 26.6, 26.7, 26.4 | 250 |  |
| *5* | *C. burnetii* | Primer (Probe: 250 nM) | 400, 500, 600, 700, 800, 900, 1000 | 26.6, 27.1, 27.0, 26.4, 26.2, 25.9, 26.5 | 900 | 25.5 |
|  |  | Probe (Primer: 900 nM) | 150, 200, 250, 300, 350 | 25.9, 25.7, 25.5, 25.4, 25.8 | 250 |  |
|  | *Ehrlichia* spp. | Primer (Probe: 250 nM) | 400, 500, 600, 700, 800, 900, 1000 | 27.7, 26.6, 26.1, 25.4, 25.9, 25.2, 25.3 | 900 | 25.4 |
|  |  | Probe (Primer: 900 nM) | 150, 200, 250, 300, 350 | 25.8, 25.5, 25.6, 25.6, 25.5 | 250 |  |
|  | *T*. *gondii* | Primer (Probe: 250 nM) | 400, 500, 600, 700, 800, 900, 1000 | 25.8, 26.1, 25.8, 26.1, 25.8, 25.6, 25.7 | 900 | 25.7 |
|  |  | Probe (Primer: 900 nM) | 150, 200, 250, 300, 350 | 25.7, 25.6, 25.3, 25.5, 25.3 | 250 |  |
| 6 | Bacteriophage MS2 | Primer (Probe: 250 nM) | 400, 500, 600, 700, 800, 900, 1000 | 27.0, 27.0, 27.0, 27.5, 27.9, 27.8, 27.2 | 600 | 27.2 |
|  |  | Probe (Primer: 600 nM) | 150, 200, 250, 300, 350 | 27.4, 27.2, 27.3, 27.0, 27.8 | 250 |  |
|  | SGLV | Primer (Probe: 250 nM) | 400, 500, 600, 700, 800, 900, 1000 | 25.6, 25.7, 25.6, 26.5, 26.2, 26.4, 26.3 | 600 | 25.9 |
|  |  | Probe (Primer: 600 nM) | 150, 200, 250, 300, 350 | 26.2, 25.8, 25.9, 25.8, 25.8 | 300 |  |
|  | *Hepatozoon* spp. / *Theileria* spp. | Primer (Probe: 250 nM) | 400, 500, 600, 700, 800, 900, 1000 | 27.1, 27.2, 26.9, 27.3, 27.2, 27.1, 26.9 | 600 | 27.4 |
|  |  | Probe (Primer: 600 nM) | 150, 200, 250, 300, 350 | 27.6, 27.4, 27.3, 26.9, 26.9 | 300 |  |
| 7 | NSDV | Primer (Probe: 250 nM) | 400, 500, 600, 700, 800, 900, 1000 | 27.3, 26.6, 26.1, 26.8, 26.9, 26.7, 27.0 | 600 | 26.6 |
|  |  | Probe (Primer: 600 nM) | 150, 200, 250, 300, 350 | 27.2, 26.9, 27.1, 27.2, 27.1 | 200 |  |
|  | TAMV | Primer (Probe: 250 nM) | 400, 500, 600, 700, 800, 900, 1000 | 25.1, 25.2, 25.4, 25.8, 25.4, 25.6, 25.4 | 500 | 25.3 |
|  |  | Probe (Primer: 500 nM) | 150, 200, 250, 300, 350 | 25.7, 25.4, 25.4, 25.5, 25.4 | 200 |  |
|  | WELV | Primer (Probe: 250 nM) | 400, 500, 600, 700, 800, 900, 1000 | 25.5, 25.6, 25.6, 25.2, 25.7, 25.3, 25.5 | 500 | 25.7 |
|  |  | Probe (Primer: 500 nM) | 150, 200, 250, 300, 350 | 25.7, 25.3, 25.6, 25.9, 25.1 | 200 |  |
| 8 | *Babesia* spp. | Primer (Probe: 250 nM) | 400, 500, 600, 700, 800, 900, 1000 | 28.6, 27.9, 27.4, 26.9, 26.6, 26.9, 26.7 | 700 | 26.8 |
|  |  | Probe (Primer: 700 nM) | 150, 200, 250, 300, 350 | 27.8, 27.6, 27.1, 27.5, 26.9 | 200 |  |
|  | *Babesia* *duncani* | Primer (Probe: 250 nM) | 400, 500, 600, 700, 800, 900, 1000 | 25.9, 25.5, 25.4, 25.5, 25.6, 25.8, 25.4 | 600 | 26.0 |
|  |  | Probe (Primer: 600 nM) | 150, 200, 250, 300, 350 | 25.8, 25.7, 25.5, 25.2, 25.1 | 200 |  |
|  | *Babesia* *microti* | Primer (Probe: 250 nM) | 400, 500, 600, 700, 800, 900, 1000 | 27.4, 27.2, 27.5, 27.3, 27.8, 27.3, 27.4 | 600 | 27.3 |
|  |  | Probe (Primer: 600 nM) | 150, 200, 250, 300, 350 | 27.4, 27.1, 27.2, 27.0, 27.0 | 250 |  |
|  | NoMV | Primer (Probe: 250 nM) | 400, 500, 600, 700, 800, 900, 1000 | 27.8, 27.4, 27.5, 27.0, 27.2, 26.6, 27.1 | 700 | 27.1 |
|  |  | Probe (Primer: 700 nM) | 150, 200, 250, 300, 350 | 27.8, 27.0, 27.6, 27.9, 28.1 | 200 |  |
|  | TcTV-1 | Primer (Probe: 250 nM) | 400, 500, 600, 700, 800, 900, 1000 | 27.3, 26.8, 26.6, 26.6, 26.8, 26.5, 26.8 | 700 | 26.8 |
|  |  | Probe (Primer: 700 nM) | 150, 200, 250, 300, 350 | 27.2, 26.9, 26.7, 26.5, 27.1 | 200 |  |

**Abbreviations**: Conc., Concentration.

**Note**: All parallel comparisons were performed in triplicate (n = 3) using plasmid standards at a fixed concentration of 10^4^ copies / reaction, data are presented as mean Cq values.

Supplementary Table S10. Analytical performance, standard curve parameters, and precision of the TBP Panel

| Assay | Pathogen | Linearity | Efficiency | Coefficient of Variation (%) | | |
| --- | --- | --- | --- | --- | --- | --- |
|  |  | (R^2^) | (E, 95% CI) | Conc Log_10_  (copies / reaction) | Repeatability | Reproducibility |
| 1 | BJNV | 0.9999 | 101.4 | 5 | 0.5 | 0.8 |
|  |  |  | (100.5 - 102.5) | 6 | 0.2 | 1.5 |
|  | JMTV | 0.9996 | 97.3 | 5 | 0.6 | 0.9 |
|  |  |  | (93.5 - 101.6) | 6 | 1.0 | 1.2 |
|  | XCV | 0.9994 | 98.1 | 5 | 0.6 | 1.2 |
|  |  |  | (94.2 - 102.6) | 6 | 1.3 | 1.2 |
| 2 | *Bartonella* spp. | 0.9997 | 98.9 | 5 | 0.5 | 1.2 |
|  |  |  | (96.6 - 101.4) | 6 | 0.9 | 1.2 |
|  | *Borrelia* spp. | 0.9996 | 97.8 | 5 | 0.6 | 0.7 |
|  |  |  | (94.1 - 101.8) | 6 | 0.9 | 1.0 |
|  | TBEV | 0.9994 | 100.4 | 5 | 0.5 | 1.3 |
|  |  |  | (95.2 - 106) | 6 | 0.9 | 1.4 |
|  | YEZV | 0.9996 | 100 | 5 | 0.5 | 0.8 |
|  |  |  | (95.9 - 104.4) | 6 | 0.9 | 1.1 |
| 3 | ALSV | 0.9994 | 104.2 | 5 | 0.7 | 1.0 |
|  |  |  | (100.2 - 109.1) | 6 | 1.0 | 1.2 |
|  | *F. tularensis* | 0.9959 | 94.7 | 5 | 0.6 | 1.2 |
|  |  |  | (85.2 - 106.5) | 6 | 0.8 | 1.2 |
|  | SFTSV | 0.9986 | 94.7 | 5 | 0.6 | 1.1 |
|  |  |  | (88.2 - 101.9) | 6 | 0.9 | 1.3 |
| 4 | *A. phagocytophilum* | 0.9991 | 98.8 | 5 | 0.5 | 0.7 |
|  |  |  | (93.2 - 105.1) | 6 | 1.3 | 1.3 |
|  | CCHFV | 0.9978 | 88.7 | 5 | 0.5 | 1.2 |
|  |  |  | (81.8 - 96.2) | 6 | 1.1 | 1.3 |
|  | *Rickettsia* spp. | 0.9982 | 98.1 | 5 | 0.4 | 0.9 |
|  |  |  | (89.8 - 106.9) | 6 | 1.2 | 1.2 |
| 5 | *C. burnetii* | 0.9961 | 102.3 | 5 | 0.7 | 1.2 |
|  |  |  | (92 - 114.9) | 6 | 1.4 | 1.6 |
|  | *Ehrlichia* spp. | 0.9986 | 94.8 | 5 | 0.6 | 1.2 |
|  |  |  | (89.1 - 101) | 6 | 1.1 | 1.2 |
|  | *T*. *gondii* | 0.9977 | 104.8 | 5 | 0.6 | 1.2 |
|  |  |  | (96.8 - 114.2) | 6 | 1.0 | 1.4 |
| 6 | SGLV | 0.9803 | 90.1 | 5 | 0.6 | 0.8 |
|  |  |  | (63.1 - 137.6) | 6 | 1.5 | 1.5 |
|  | *Hepatozoon* spp. / *Theileria* spp. | 0.9942 | 96.6 | 5 | 0.6 | 1.1 |
|  |  |  | (79.4 - 116.5) | 6 | 1.5 | 1.5 |
| 7 | NSDV | 0.9950 | 99.1 | 5 | 0.7 | 0.9 |
|  |  |  | (86.8 - 114.8) | 6 | 1.2 | 1.5 |
|  | TAMV | 0.993 | 98.6 | 5 | 0.7 | 1.2 |
|  |  |  | (75.4 - 122.3) | 6 | 1.2 | 1.2 |
|  | WELV | 0.9951 | 94.8 | 5 | 0.7 | 1.1 |
|  |  |  | (84.1 - 109.2) | 6 | 1.5 | 1.7 |
| 8 | *Babesia* spp.^†^ | 0.9994 | 103.7 | 5 | 0.5 | 1.3 |
|  |  |  | (98.4 - 109.5) | 6 | 0.8 | 1.6 |
|  | *Babesia duncani*^†^ | 0.9991 | 98.8 | 5 | 0.4 | 1.0 |
|  |  |  | (93 - 105.3) | 6 | 0.9 | 1.4 |
|  | *Babesia microti*^†^ | 0.9992 | 106.9 | 5 | 0.5 | 0.7 |
|  |  |  | (100.9 - 113.6) | 6 | 1.2 | 1.0 |
|  | NoMV | 0.9995 | 98.6 | 5 | 0.6 | 1.5 |
|  |  |  | (94.9 - 102.5) | 6 | 1.1 | 1.7 |
|  | TcTV-1 | 0.9994 | 99.7 | 5 | 0.4 | 1.0 |
|  |  |  | (95.3 - 104.4) | 6 | 1.1 | 1.7 |

**Abbreviations**: Conc Log_10_, Concentration logarithm 10; Rep, Repeatability (intra-assay precision); Repr, Reproducibility (inter-assay precision).

Supplementary Table S11. Performance analysis of dPCR and its sensitivity comparison with TaqMan qPCR using plasmid standard

| Assay | Pathogen | LOB (Digital PCR)  (copies / reaction) | LOD_99%_ (99% CI, copies / reaction) | |
| --- | --- | --- | --- | --- |
|  |  |  | Digital PCR | TaqMan qPCR |
| 1 | BJNV | 8.4 | 58 (45.0 - 104.1) | 16 (13.6 - 30.4) |
|  | XCV | 0 | 17.2 (13.6 - 29.9) | 8.1 (6.7 - 18.2) |
|  | JMTV | 2.5 | 18.5 (14.4 - 48.9) | 13.5 (10.9 - 22.3) |
| 2 | *Bartonella* spp. | 2.7 | 22.8 (15.7 - 83.2) | 65.8 (53.5 - 106.1) |
|  | *Borrelia* spp. | 0 | 32.6 (22.4 - 118.9) | 82.3 (65.9 - 126) |
|  | TBEV | 2.6 | 7.9 (6.7 - 20.9) | 131 (106.8 - 305.1) |
|  | YEZV | 0 | 19 (13.5 - 47.3) | 26 (20.1 - 52) |
| 3 | Alongshan | 0 | 11.5 (8.7 - 36.5) | 3.5 (3 - 7) |
|  | *F*. *tularensis* | 8.4 | 13 (8.7 - 38.7) | 10.9 (6.9 - 67.6) |
|  | SFTSV | 6.8 | 35.4 (24.5 - 160.8) | 10.9 (9 - 21.4) |
| 4 | *A*. *phagocytophilum* | 0 | 38.1 (28.8 - 112.8) | 55.9 (41.7 - 242.7) |
|  | CCHFV | 0 | 27.2 (23.3 - 48.8) | 68.9 (43.4 - 246.4) |
|  | *Rickettsia* spp. | 0 | 33.9 (25.5 - 102.6) | 89.5 (65.4 - 181.1) |
| 5 | *C*. *burnetii* | 0 | 15.4 (13.8 - 23.1) | 42.5 (37.3 - 183) |
|  | *Ehrlichia* spp. | 0 | 6.2 (5.5 - 9.3) | 87.1 (66.1 - 186.2) |
|  | *T*. *gondii* | 0 | 16.6 (14.0 - 37.4) | 119.2 (97.2 - 320.3) |
| 6 | SGLV | 5.8 | 14.5 (11.7 - 67.3) | 107.8 (81.7 - 213.6) |
|  | *Hepatozoon* spp. / *Theileria* spp. | 4.6 | 13.2 (10.6 - 62.2) | 68.1 (51.8 - 133.5) |
| 7 | NSDV | 0 | 28.8 (20.8 - 130.3) | 4.4 (4.1 - 7.1) |
|  | TAMV | 0 | 28.3 (20.5 - 127.9) | 11.6 (10.1 - 16.7) |
|  | WELV | 2.5 | 30.3 (22.6 - 141.5) | 6.4 (6 - 8.9) |
| 8 | *Babesia* spp. | 5.7 | 25.8 (19.4 - 27.1) | 41.3 (32.8 - 81.4) |
|  | *Babesia* *duncani* | 0 | 2.2 (2- 3.9) | 21.7 (17.8 - 48.1) |
|  | *Babesia* *microti* | 0 | 14.5 (10.1 - 129.3) | 5.9 (5.3 - 9) |
|  | NoMV | 0 | 13 (9.5 - 33.8) | 5 (4.5 - 8) |
|  | TcTV-1 | 0 | 5 (4.4 - 24.5) | 7 (6.1 - 18.6) |

**Abbreviations**: LOB, Limit of Blank; LOD, Limit of Detection.

Supplementary Table S12. Discrepancy analysis and confirmatory testing results for 35 discordant samples identified during multiplex qPCR validation

| No. | Target Pathogen | qPCR (Cq) | nPCR+Sequencing | dPCR (copies/µL) | Final Consensus Status |
| --- | --- | --- | --- | --- | --- |
| 1 | *Bartonella* spp. | Positive (38.0) | Negative | Negative | False Positive |
| 2 | *Bartonella* spp. | Positive (38.0) | Negative | Negative | False Positive |
| 3 | *Bartonella* spp. | Negative (40.0) | Positive | ND | False Negative |
| 4 | *Borrelia* spp. | Positive (38.0) | Negative | Negative | False Positive |
| 5 | *Borrelia* spp. | Positive (38.0) | Negative | Negative | False Positive |
| 6 | *C. burnetii* | Positive (33.3) | Negative | Positive (1.9) | True Positive |
| 7 | *C. burnetii* | Positive (34.5) | Negative | Positive (0.8) | True Positive |
| 8 | *Ehrlichia* spp. | Positive (36.0) | Negative | Positive (16.6) | True Positive |
| 9 | *Ehrlichia* spp. | Positive (33.7) | Negative | Positive (55.5) | True Positive |
| 10 | *Ehrlichia* spp. | Positive (37.8) | Negative | Negative | False Positive |
| 11 | *Ehrlichia* spp. | Positive (37.8) | Negative | Negative | False Positive |
| 12 | *Ehrlichia* spp. | Positive (38.0) | Negative | Negative | False Positive |
| 13 | *Hepatozoon* spp. */ Theileria* spp. | Positive (33.0) | Negative | Positive (30.5) | True Positive |
| 14 | *Hepatozoon* spp. */ Theileria* spp. | Positive (38.3) | Negative | Positive (5.7) | True Positive |
| 15 | *Hepatozoon* spp. */ Theileria* spp. | Positive (38.0) | Negative | Negative | False Positive |
| 16 | NoMV | Negative (40.0) | Positive | ND | False Negative |
| 17 | NSDV | Positive (38.3) | Negative | Positive (4.5) | True Positive |
| 18 | NSDV | Positive (36.3) | Negative | Negative | False Positive |
| 19 | NSDV | Positive (36.8) | Negative | Negative | False Positive |
| 20 | NSDV | Positive (36.8) | Negative | Negative | False Positive |
| 21 | *Rickettsia* spp. | Positive (35.9) | Negative | Positive (0.4) | True Positive |
| 22 | *Rickettsia* spp. | Positive (33.8) | Negative | Positive (1.5) | True Positive |
| 23 | *Rickettsia* spp. | Positive (37.1) | Negative | Negative | False Positive |
| 24 | *Rickettsia* spp. | Positive (37.5) | Negative | Negative | False Positive |
| 25 | *Rickettsia* spp. | Positive (37.6) | Negative | Negative | False Positive |
| 26 | *Rickettsia* spp. | Positive (37.7) | Negative | Negative | False Positive |
| 27 | *Rickettsia* spp. | Negative (40.0) | Positive | ND | False Negative |
| 28 | *Rickettsia* spp. | Negative (40.0) | Positive | ND | False Negative |
| 29 | SFTSV | Positive (38.8) | Negative | Negative | False Positive |
| 30 | SFTSV | Positive (36.9) | Negative | Negative | False Positive |
| 31 | SFTSV | Positive (37.6) | Negative | Negative | False Positive |
| 32 | SGLV | Positive (38.0) | Negative | Negative | False Positive |
| 33 | SGLV | Positive (38.4) | Negative | Negative | False Positive |
| 34 | WELV | Positive (38.7) | Negative | Negative | False Positive |
| 35 | XCV | Positive (38.2) | Negative | Negative | False Positive |

**Abbreviations:** ND, not determined.

Supplementary Table S13. Detection of pathogens across various rodent samples

| Type | *Bartonella* spp. (%) | *Borrilia* spp. (%) | *Ehrlichia* spp. (%) | *Rickettsia* spp. (%) |
| --- | --- | --- | --- | --- |
| *Niviventer fulvescens* | 1/1 (100) | 0/1 (0) | 0/1 (0) | 0/1 (0) |
| *Niviventer confucianus* | 4/4 (100) | 0/4 (0) | 0/4 (0) | 0/4 (0) |
| *Rattus andamanensis* | 5/7 (57.1) | 1/7 (14.3) | 1/7 (14.3) | 0/7 (0) |
| *Rattus rattus* | 0/2 (0) | 0/2 (0) | 0/2 (0) | 0/2 (0) |
| *Rattus tanezumi* | 29/45 (64.4) | 3/45 (6.7) | 0/45 (0) | 3/45 (6.7) |
| Total | 39/59 (67.8) | 4/59 (6.8) | 1/59 (1.7) | 3/59 (5.1) |

Supplementary Figure S1. Representative 1D fluorescence amplitude plots of the dPCR assay for 25 tick-borne pathogens

**Notes:** A-H display the dPCR results for pathogens grouped in assay 1-8, respectively. Each plot visualizes the successful partitioning and robust signal separation achieved on the QIAcuity nanoplates. Target-positive partitions (blue) are clearly resolved from the baseline negative population (grey), demonstrating a high signal-to-noise ratio across all assays.
